# Ultra‐Long Lived Luminescent Triplet Excited States in Cyclic (Alkyl)(amino)carbene Complexes of Zn(II) Halides

**DOI:** 10.1002/chem.202201114

**Published:** 2022-06-21

**Authors:** Ondřej Mrózek, Markus Gernert, Andrey Belyaev, Mousree Mitra, Lars Janiak, Christel M. Marian, Andreas Steffen

**Affiliations:** ^1^ Faculty of Chemistry and Chemical Biology TU Dortmund University Otto-Hahn-Str. 6 44227 Dortmund Germany; ^2^ Institute of Theoretical and Computational Chemistry Heinrich Heine University Düsseldorf 40225 Düsseldorf Germany

**Keywords:** carbenes, DFT/MRCI, luminescence, phosphorescence, zinc

## Abstract

The high element abundance and d^10^ electron configuration make Zn^II^‐based compounds attractive candidates for the development of novel photoactive molecules. Although a large library of purely fluorescent compounds exists, emission involving triplet excited states is a rare phenomenon for zinc complexes. We have investigated the photophysical and ‐chemical properties of a series of dimeric and monomeric Zn^II^ halide complexes bearing a cyclic (alkyl)(amino)carbene (cAAC) as chromophore unit. Specifically, [(cAAC)XZn(μ‐X)_2_ZnX(cAAC)] (X=Cl (**1**), Br (**2**), I (**3**)) and [ZnX_2_(cAAC)(NCMe)] (X=Br (**4**), I (**5**)) were isolated and fully characterized, showing intense visible light photoluminescence under UV irradiation at 297 K and fast photo‐induced transformation. At 77 K, the compounds exhibit improved stability allowing to record ultra‐long lifetimes in the millisecond regime. DFT/MRCI calculations confirm that the emission stems from ^3^XCT/LE_cAAC_ states and indicate the phototransformation to be related to asymmetric distortion of the complexes by cAAC ligand rotation. This study enhances our understanding of the excited state properties for future development and application of new classes of Zn^II^ phosphorescent complexes.

## Graphical abstract

Zinc(II) halide cAAC dimers undergo photochemically induced rearrangements in the triplet excited state, one of which involves rotation of one of the cAAC ligands giving an asymmetric dimeric structure at 77 K. Consequently, transformation of ^3^XCT/LE to purely ^3^LE states localized at the carbene lead to ultra‐long lived phosphorescence (millisecond regime). At room temperature, further rearrangements are observed. Similar photoreactions occur in monomeric [ZnX_2_(cAAC)(MeCN)] complexes.

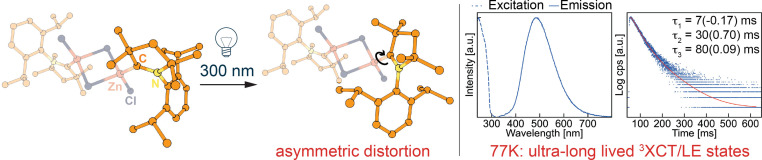

## Introduction

Photoactive transition metal (TM) complexes provide the opportunity to exploit the properties of triplet excited states in manifold applications, such as photocatalysis, photodynamic therapy, bioimaging and (organic) light emitting devices (OLEDs).[^1^, ^2^, ^3^, ^4^] The benefit of the TM centre in comparison to purely organic systems is usually attributed to its potential to facilitate spin‐forbidden processes, such as intersystem crossing (ISC) S_n_→T_n_ or phosphorescence T_1_→S_0_, by mediating spin‐orbit coupling (SOC) between excited states of different multiplicity.^[5]^ The SOC constant strongly depends on the effective nuclear charge and, consequently, research in the above mentioned application areas has mostly been focussed on complexes of 4d and 5d heavy metals, for example, Ir^III^, Pt^II^ or Ru^II^. The success of this strategy has led to tremendous scientific and technological progress, involving utilization of such compounds in commercial second‐generation (phosphorescence‐based) OLEDs[^6^, ^7^] or as industrial high‐efficiency photocatalysts.[^1^, ^8^]

Although 3d TM complexes in general suffer from much weaker operative SOC in comparison to their heavier congeners, which might hamper ISC processes, significant attention has been devoted to molecular systems based on metal cations with closed‐shell (3d^0^ or 3d^10^) electron configurations.[^9^, ^10^, ^11^, ^12^] Many factors triggered research in this direction, such as high element abundance, their variable coordination geometries, redox properties, absence of metal‐centred d‐d* states that might induce fast non‐radiative decay, and the discovery of thermally activated delayed fluorescence (TADF). The latter process is particularly useful to harvest triplet excitons by reverse ISC T_n_→S_1_ and subsequent fluorescence, bypassing the spin‐forbidden phosphorescence and reducing the importance of SOC. This mechanism is often found in Cu^I^ complexes with a donor‐metal‐acceptor structure, and it has been shown that up to 100 % quantum yield and (sub‐)microsecond lifetimes can be achieved, e. g. in cyclic (alkyl)(amino)carbene (cAAC)[^13^, ^14^]/amide compounds.[^15^, ^16^] The resulting radiative rate constants *k*_r_ of up to 10^5^–10^6^ s^−1^ are competitive and, in some cases, even faster compared to the best heavy transition metals emitters.

**Scheme 1:**
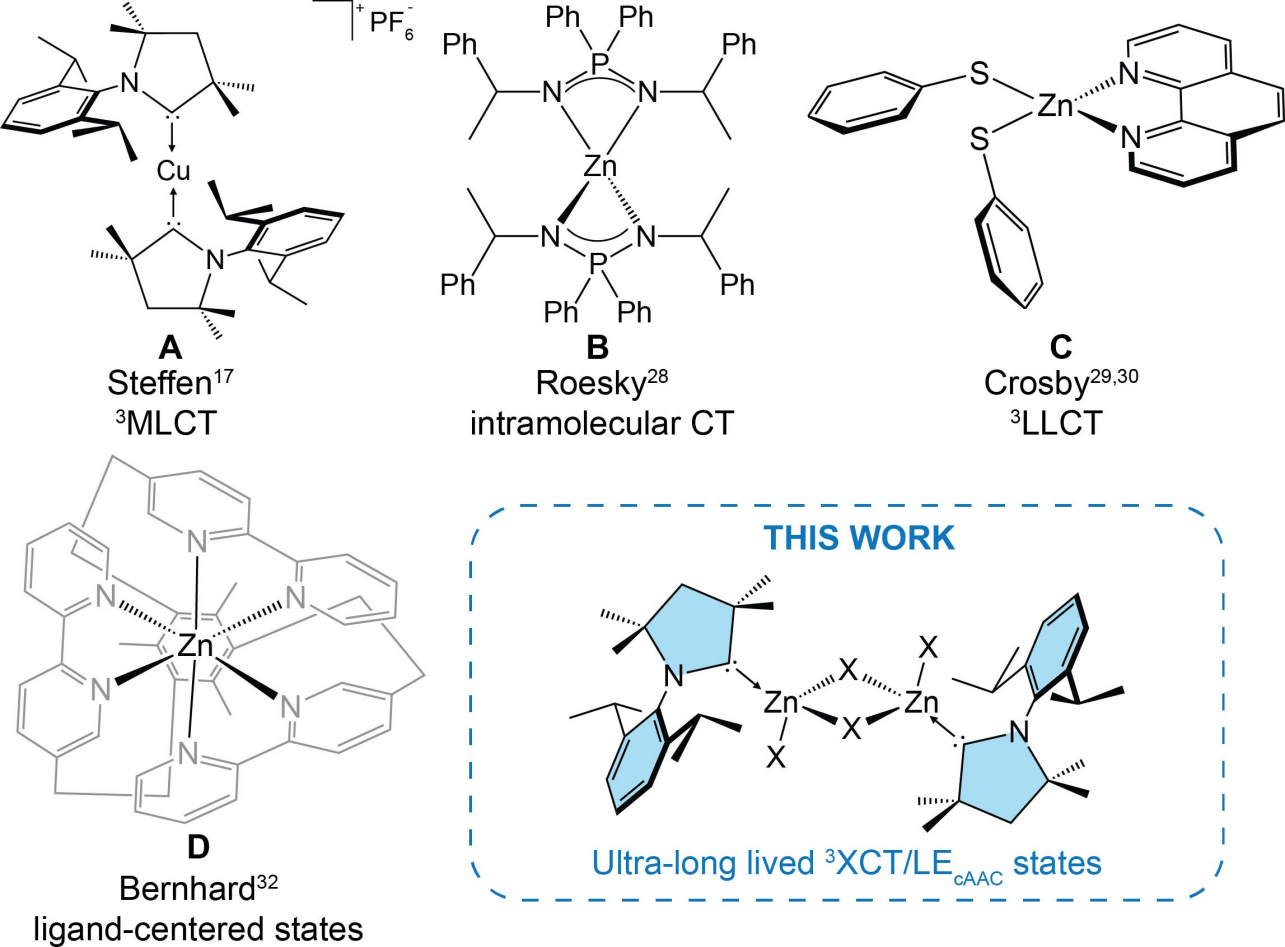
Selected examples of phosphorescent and TADF complexes of Cu(I) and Zn(II) reported in literature.

However, judicious choice of ligands in combination with the low energy oxidation of Cu^I^ also allow to greatly increase the MLCT character and thus the operative SOC of the excited states, which can also enhance *k*_r_. For example, the very strong σ‐donating and π‐accepting properties of the cAAC ligands in homoleptic [Cu(^Me^cAAC)_2_][PF_6_] (Scheme 1A) lead to destabilization of the HOMOs with pronounced d‐orbital character, whereas the LUMO (π* character) orbital is stabilized.^[17]^ As a result, the T_1_ state is strongly coupled to singlet excited states with high oscillator strength, leading to *k*_r_ of 9 x 10^4^ s^−1^, which is the highest value reported so far for purely phosphorescent Cu^I^ compounds.

Interestingly, despite the d^10^ electron configuration of Zn^II^ and its high abundance (∼75 ppm in the Earth's crust), only few triplet emitters have been developed so far, but a large number of purely fluorescent Zn^II^ complexes is known.[^18^, ^19^, ^20^, ^21^, ^22^, ^23^, ^24^, ^25^, ^26^, ^27^, ^28^] This may be due to the fact that MLCT states cannot be formed due to the much higher ionization energy of Zn^II^ compared to Cu^I^, which precludes the metal d‐orbitals to be involved in the excited states and mediate SOC. Thus, predominantly intra‐ligand CT (ILCT) or LC states have been studied for phosphorescence or TADF.

For instance, Adachi et al.^[25]^ described a Zn^II^ TADF emitter by employing ligands with a donor‐acceptor motif. The significant spatial separation of the HOMO and LUMO within the ligand scaffold led to a small singlet‐triplet energy gap, and external quantum efficiencies of up to 20 % were achieved when applied in OLEDs. Roesky et al. recently reported Zn^II^ iminophosphonamide (NPN) complexes (Scheme 1B) displaying delayed fluorescence in the solid state.^[28]^ In this case, twisting of the NPN units in the T_1_ state is the mechanism that supports the thermally driven spin flip to the singlet excited state as the parent compound with planar ligand arrangement showed prompt fluorescence.^[28]^ However, the quantum yields are relatively low (*ϕ*<0.05) at 297 K, and thus this study nicely pinpoints the challenges and obstacles to overcome for the design of efficient Zn^II^‐based triplet emitters.

In contrast to TADF, phosphorescence is a surprisingly rare phenomenon for zinc‐based compounds, except for porphyrins. Back in 1985, Crosby et al. reported an intriguing photophysical behaviour of [Zn(SR’)_2_(phen)] type complexes (phen=1,10‐phenanthroline, SR’=various thiophenolate derivatives) (Scheme 1C).[^29^, ^30^] The T_1_ state of LLCT character is populated via internal conversion from an energetically higher lying ^3^ππ* state, that is associated with an energetic barrier.[^29^, ^30^] However, the main limitation is dominant non‐radiative relaxation at room temperature, leading to efficient emission only at low temperatures. Yam and co‐workers further modified the phen ligand by introducing dithienyl moieties in the 1,10‐positions to design a series of photoswitchable compounds that emit from ^3^LLCT states at 298 K.^[31]^ A rigid octahedral (so‐called hemi‐cage) complex bearing three bipyridine units connected by a mesityl cap (Scheme 1D) has also been reported to phosphoresce, in contrast to the fluorescent compound [Zn(bpy)_3_][PF_6_]_2_ (bpy=2,2’‐bipyridine). The switch of emission occurring from the S_1_ or T_1_ state was associated with the hexa‐alkyl‐substituted benzene fragment, of which the authors argue to introduce a low‐energy triplet state of ligand‐centred (LC) character.^[32]^ However, our own experimental and theoretical studies on these compounds that we will report in due course suggest the emission to be fluorescence in nature instead of phosphorescence for both compounds.

In order to broaden the horizon of photoactive 3d TM complexes, we were interested in the investigation of zinc compounds featuring LLCT states, which have received only very limited attention so far. Considering their strong excited state π‐acceptor properties, cAACs seem to be suitable ligands for the development of donor‐Zn^II^‐acceptor compounds. Herein, we report on the synthesis, characterization and photophysical study of ^Me^cAAC complexes of ZnX_2_ (X=Cl, Br, I) (Scheme 1). The experimental study revealed dimeric character of ZnX_2_:^Me^cAAC adducts when crystallized from THF. In addition, these complexes show fluxional behaviour in CH_3_CN solution, allowing the isolation of the monomeric complexes [ZnX_2_(^Me^cAAC)(NCCH_3_)] for X=Br and I. Both monomeric and dimeric species undergo photo‐induced transformations upon UV light irradiation, forming ultra‐long‐lived triplet excited states of ^3^LLCT character. These first experimental and DFT/MRCI studies on Zn^II^ complexes bearing cAAC ligands as π‐chromophores indicate great potential of this compound class for the design of long‐lived states for future applications.

## Results and Discussion

### Synthesis and characterization

**Scheme 2:**
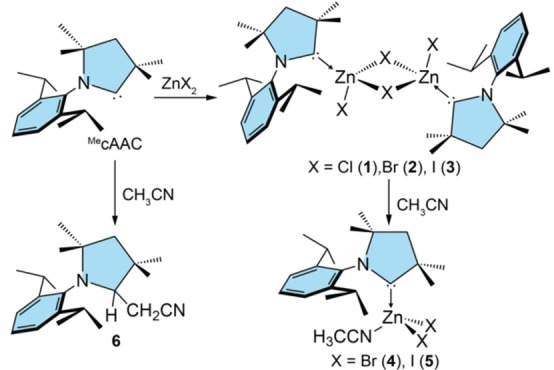
Synthesis of the dimeric complexes **1**–**3** and their subsequent reactivity towards CH_3_CN giving **4** and **5**, and reaction of ^Me^cAAC with CH_3_CN.

**Figure 1:**
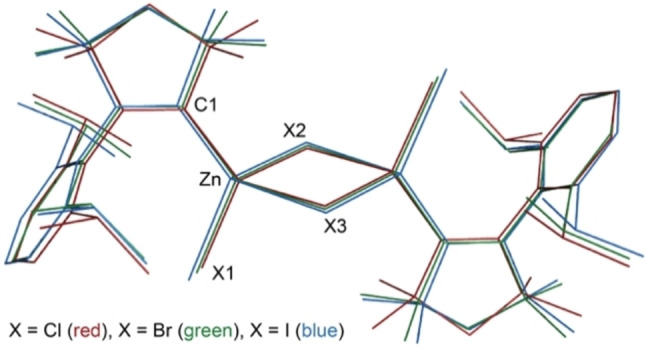
Superimposed sets of coordinates obtained by X‐ray diffraction analysis for **1** (red), **2** (green) and **3** (blue). Hydrogen atoms are omitted for clarity. Selected bond lengths (Å) and angles (°) for **1** (X=Cl), **2** (X=Br) and **3** (X=I): Zn−X1 2.2119(5), 2.3533(3), 2.5559(5); Zn−X2 2.3925(6), 2.5299(4), 2.7504(4); Zn−X3 2.3830(5), 2.5233(4), 2.7208(5); Zn−C1 2.0547(17), 2.0547(13), 2.069(3); X1−Zn−C1 123.79(5), 124.72(4), 125.57(8); X1−Zn−X2 106.61(2), 104.76(1), 103.28(1); X1−Zn−X3 105.94(2), 106.13(1), 105.57(1); C1−Zn−X2 110.86(5), 112.26(4), 111.09(8); C1−Zn−X3 113.20(5), 111.05(4), 112.06(8); X2−Zn−X3 91.50(2), 93.19(1), 94.61(2).

Our investigation of Zn^II^ carbene complexes was inspired by a reaction reported by Roesky et al., who suggested that mixing of ZnCl_2_ and ^Me^cAAC in THF at −50 °C would afford the monomeric adduct ^Me^cAAC:ZnCl_2_ according to NMR spectroscopic studies.^[33]^ We further optimized the reaction conditions for its isolation and found that mixing of pre‐cooled (−40 °C) THF solutions of ZnCl_2_ and ^Me^cAAC, respectively, and slow warming to room temperature over 2 h is a reliable and reproducible method for preparation of single crystals suitable for X‐ray diffraction on up to 200 mg scale (Scheme 2). Instead of a monomer, a dimeric arrangement [(^Me^cAAC)ClZn(μ‐Cl)_2_ZnCl(^Me^cAAC)] (**1**) was isolated, which is a more intuitive outcome considering the preference of zinc(II) for a tetrahedral coordination geometry (Figure 1). In the case of ZnBr_2_ and ZnI_2_, slow diffusion of *n*‐pentane into the reaction mixture also provided single crystals that showed analogous dimeric configurations [(^Me^cAAC)XZn(μ‐X)_2_ZnX(^Me^cAAC)] (X=Br (**2**), X=I (**3**)). Importantly, once crystallized, complexes **2** and **3** are not resoluble in THF, which suggests initial formation of more soluble trigonal planar or, more likely, THF‐coordinated monomers in the reaction mixtures. Unfortunately, all attempts to isolate these postulated species failed as every tested crystallization technique (low‐temperature crystallization, slow evaporation of THF solvent or diffusion of various anti‐solvents) always afforded the dimers **2** and **3**. Compounds **1**–**3** are highly sensitive towards air and moisture, which requires further handling also for the photophysics under inert atmosphere. During the synthesis of **1**–**3**, we further noted the formation of 1–3 % of by‐products involving protonation of the carbene ligand, which were identified as ^Me^cAAC(H)[ZnCl_3_] and (^Me^cAAC(H))_2_[ZnCl_4_] (for X‐ray data see Supporting Information). Fortunately, both compounds are easily washed from our target dimeric complexes using THF.

The structures of **1**–**3** in the single‐crystalline solid state can be viewed as two pseudo‐tetrahedral {(^Me^cAAC)Zn(μ‐X)_2_X} units sharing one edge consisting of two bridging halides, with overall approximate D_*2*_ symmetry. Only a few analogous Zn^II^ carbene complexes have been reported in the literature to compare the structural parameters, which are [(IPr)BrZn(μ‐Br)_2_ZnBr(IPr)]^[34]^ (IPr=1,3‐dimesityl‐imidazol‐4,5‐dihydro‐2‐ylidene), [(IPr’)ClZn(μ‐Cl)_2_ZnCl(IPr’)]^[35]^ (IPr’=1‐mesityl‐3‐(methoxyethanyl)‐imidazol‐4,5‐dihydro‐2‐ylidene) and [(BIcAAC)ClZn(μ‐Cl)_2_ZnCl(BIcAAC)] (BIcAAC=bicyclic (alkyl)(amino)carbene).^[36]^ The zinc−halide bond lengths progressively increase in a row Cl > Br > I (Figure 1), whereas the Zn−C^carbene^ bonds of **1**–**3** show only minor variations and are very similar to those found for the NHC‐ and BIcAAC‐based dimers mentioned above (∼2.0 Å).[^34^, ^35^, ^36^]

Dimers **1**–**3** are not soluble in aliphatic or aromatic hydrocarbons, or chlorinated solvents; however, they could be redissolved in acetonitrile for further characterization by multinuclear NMR spectroscopy (see Experimental Section). Coordination of the ^Me^cAAC ligand to the Zn^II^ ion leads to a significant upfield shift of the C^carbene^ resonance in the ^13^C{^1^H} NMR spectra in CD_3_CN to ∼250 ppm for **1**–**3** compared to 304.2 ppm of free ^Me^cAAC.^[14]^ The chemical shift of the ^15^N resonance is a useful tool to analyse the degree of σ‐donation and π‐backbonding in the metal−carbene bond.^[37]^ For complexes with dominant carbene→M σ‐donating contribution, the nitrogen resonates at around –160 ppm, whereas for species with increased π‐backbonding, the signal is considerably upfield shifted up to −300 ppm.^[37]^ In the case of **1**–**3**, the ^15^N resonances experience a very high chemical shift of ca. −146 ppm, which suggests almost pure σ‐donor character of the ^Me^cAAC−Zn bond as expected considering the dicationic charge of the metal ion. The MALDI mass spectra (MS) of **1**–**3** revealed extensive fragmentation (Figure S9–S15) despite a mild ionization, including cleavage of both Zn−X and Zn−C^carbene^ bonds, producing cationic fragments [(^Me^cAAC)ZnX]^+^ (m/z=430 (X=Br), m/z=476 (X=I)) and organic ions related to the carbene moiety, ^Me^cAACH^+^ (m/z=286). In the case of **1**, the spectrum also shows an MS peak with m/z=807. This fragment was assigned to dimeric compounds with one abstracted chloride, [(^Me^cAAC)_2_Zn_2_(Cl)_3_]^+^.

**Figure 2:**
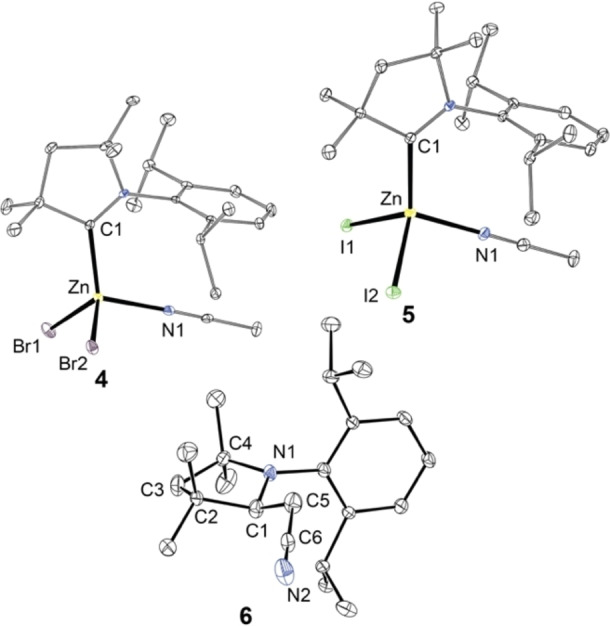
ORTEP view of compounds **4**–**6**. Thermal ellipsoids are drawn at the 30 % probability level and hydrogens are omitted for clarity. Selected bond lengths (Å) and angles (°) for **4** (X=Br) and **5** (X=I): Zn−X1 2.3942(7), 2.6290(5); Zn−X2 2.3957(6), 2.5957(8); Zn−N1 2.072(2), 2.009(3); Zn−C1 2.047(3), 2.069(3); X1−Zn−X2 110.98(2), 106.68(2); X1−Zn−N1 101.26(6), 101.84(9); X2−Zn−N1 104.85(5), 101.14(9); N1−Zn−C1 111.07(9), 112.48; X1−Zn−C1 119.18(7), 114.97(8); X2−Zn−C1 104.85(5), 117.73(9).

**Figure 3:**
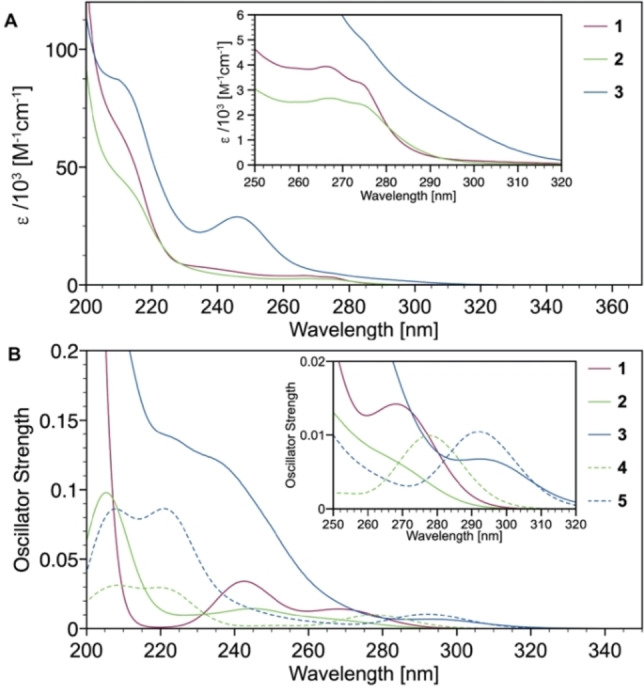
Experimental (**A**) and calculated (**B**) absorption spectra of complexes **1**–**5** in MeCN solution. The simulated spectra of compounds 1–5 are displayed which were obtained by broadening the spin−orbit coupled DFT/MRCI line spectra by Gaussian functions of 1200 cm^−1^ FWHM.

For compound **1**, crystallization from CH_3_CN/Et_2_O mixture gave the same dimeric arrangement. However, under the same conditions, the lower Zn−X bond dissociation energies of **2** and **3** lead to a higher tendency for monomerization upon solvent coordination and [ZnX_2_(^Me^cAAC)(NCCH_3_)] (X=Br (**4**), I (**5**)) (Scheme 1) were isolated, which are also sensitive to ambient environment as found for the dimers. The Zn−X interatomic distances and Zn−C^carbene^ bonds of **4** and **5** have almost the same values as determined for **2** and **3** (compare Figures 2 and 3), and the NMR spectra in CD_3_CN of the respective halide derivatives are identical. We note that the ^1^H NMR spectrum contains an additional resonance with an integral intensity of ∼3 at ∂=1.96 ppm corresponding to free CH_3_CN.^[38]^ Although simple ligand exchange of CH_3_CN for CD_3_CN might be responsible for the liberation of coordinated acetonitrile, photophysical measurements (see below) suggest that backward dimerization to **2** and **3** might take place in solution.

All attempts to induce analogous coordination of CH_3_CN and subsequent monomerization of the chlorido derivative **1** by increasing the reaction temperature led to progressive conversion to a mixture of compounds, including ^Me^cAAC(H)[ZnCl_3_] (also one of the minor by‐products of the synthesis of **1**, see above) and the C−H activation product of acetonitrile by the free carbene, ^Me^cAAC(H)CH_2_CN (**6**). Although it is unclear what species acts as a proton source for ^Me^cAAC, these decomposition reactions of the zinc(II) complex **1** probably occur by initial dissociation of ^Me^cAAC. A similar process, including protonation of the carbene with residual water, has been reported for [CuX_2_(IDipp)] (IDipp=1,3‐bis(2,6‐diisopropylphenyl)imidazol‐2‐ylidene).^[39]^ In addition, free ^Me^cAAC is capable of C−H activation of toluene at elevated temperatures,^[40]^ which makes the non‐metal‐mediated addition of more reactive acetonitrile to the free carbene quite likely. To prove this hypothesis, we reacted free ^Me^cAAC with dry CH_3_CN and indeed found quantitative conversion to **6** (see Supporting Information), for which we confirmed its identity by single‐crystal X‐ray diffraction (Figure 2).

### Photophysical and DFT/MRCI studies

The absorption spectra of **1** and **2** in acetonitrile are very similar in their appearance, with intense bands in the UV between *λ*=200‐230 nm and extinction coefficients of *ϵ* = ca. 25–100×10^3^ M^−1^ cm^−1^ that we tentatively assign to LC transitions of the ^Me^cAAC ligand (Figure 3A).

**Figure 4:**
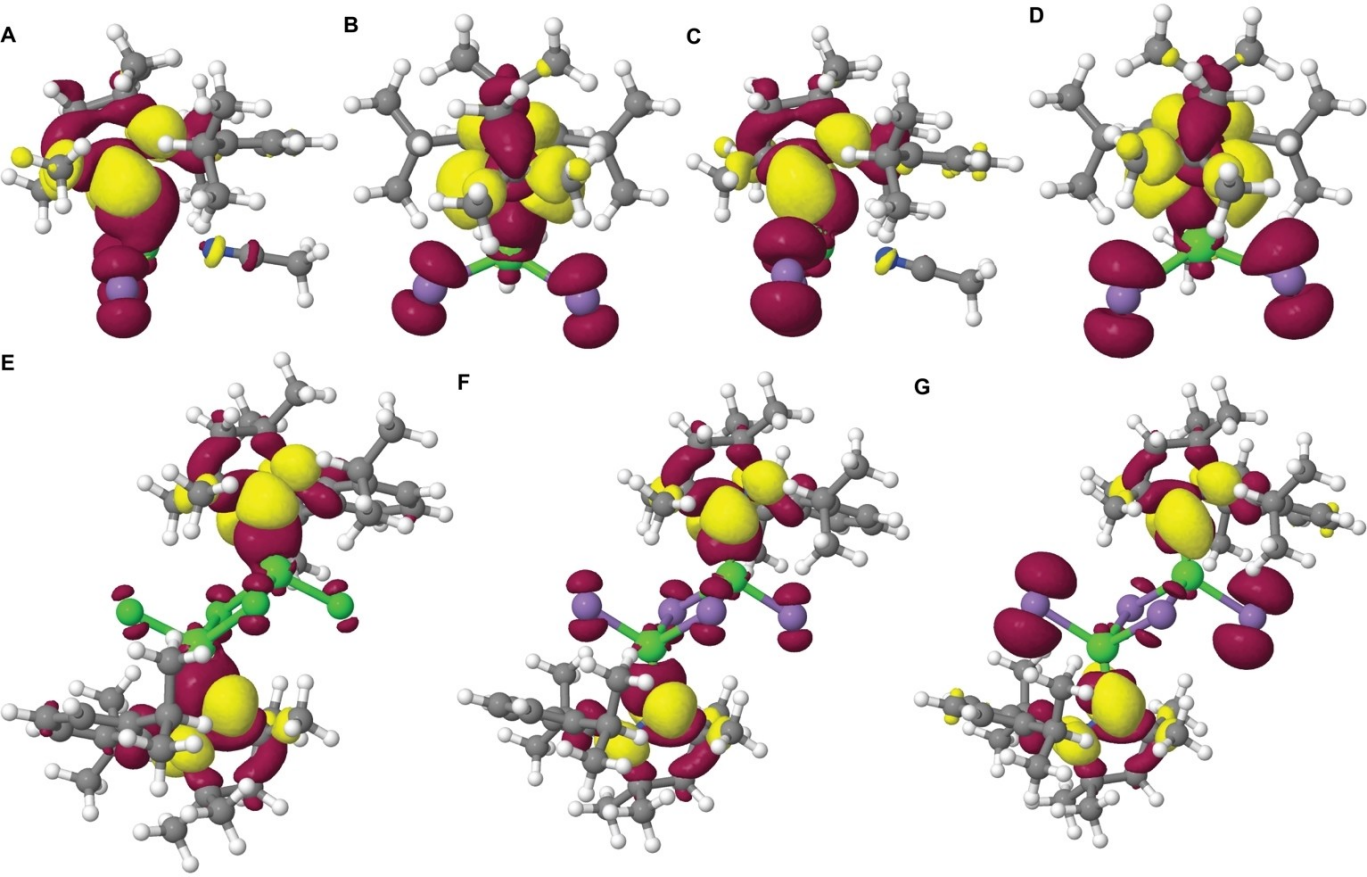
Difference densities for the S_0_→S_1_ transitions of complex **4** (A: top view, B: side view), complex **5** (C: top view, D: side view), and dimers **1** (E), **2** (F) and **3** (G) at the respective ground‐state geometries. Areas losing electron density upon absorption are shown in red, areas gaining electron density in yellow

Much weaker low‐energy absorptions between *λ*=250−280 nm with *ϵ*=2,800‐4,200 M^−1^cm^−1^ originate from in‐plane σ‐type to out‐of‐plane π*‐type transitions, with some minor contribution from the halides. The respective S_0_→S_1u_ excitations depicted in Figure 4 are similarly structured as recently reported for the homoleptic complex [Cu(^Me^cAAC)_2_][PF_6_].^[17]^ The iodo‐bridged dimer **3** shows comparable spectral absorption features, but with much higher oscillator strength at *λ*=255 nm with *ϵ*=30×10^3^ M^−1^cm^−1^ and a very broad, bathochromically shifted lowest energy absorption band between *λ*=280–320 nm. We note that the participation of the terminal halide ligands in particular in the first transition increases in the series Cl<Br<I (Figure 4). Together with the enhanced singlet‐triplet admixture in the iodo complex **3** due to strong SOC, the larger XCT contribution explains the marked redshift of the S_0_→S_1u_ band observed in both, experiment, and theory.

The experimental absorption spectra upon dissolving single‐crystalline samples of monomeric [ZnX_2_(^Me^cAAC)(NCCH_3_)] (X=Br (**4**), I (**5**)) in MeCN are identical to those of the respective dimers **2** and **3** (Figures S17 and S18), although our DFT/MRCI studies predict non‐structured characteristic bands at *λ*=278 (**4**) and 291 nm (**5**) (Figure 3B). These low energy bands would originate from XCT/LE transitions, that involve mainly the p orbitals of the halides and in‐plane σ‐type orbitals of the carbene as donors, and the C‐N antibonding π*(cAAC) orbital as acceptor (Figure 4A–D). In addition, there are significant differences between the calculated oscillator strengths for **4**/**5** and the observed respective experimental absorption intensities in the region *λ*=240‐260 nm, that match those of **2** and **3**, respectively. Overall, these findings are likely caused by backward dimerization of **4** and **5** to structures **2** and **3** in MeCN solution.

**Figure 5:**
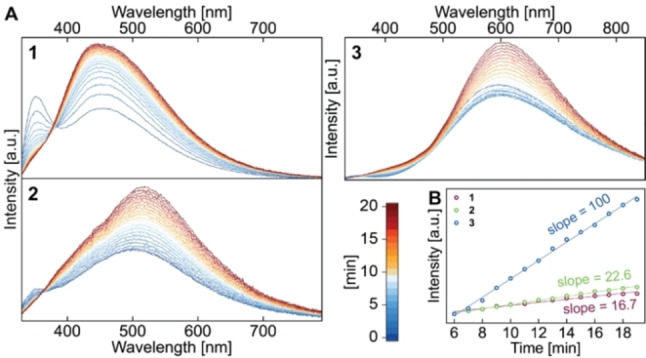
Multiple emission spectra recorded for **1**–**3** in solid‐state at room temperature (**A**) and emission intensity‐time plot (**B**) recorded at *λ*_max_=451 (**1**), 515 (**2**) and 570 (**3**) nm.

In the single‐crystalline solid‐state, the dimers **1**–**3** show dual photoluminescence when irradiated at *λ*_ex_=300 nm, with a high energy band at *λ*_max_=360 (**1**), 370 (**2**) and 355 (**3**) nm, and a second, very broad low energy band (*λ*_max_=451 (**1**), 515 (**2**) and 570 (**3**) nm) (Figure 5A). The ratio of the two bands strongly depends on the halide, with the chloride complex **1** featuring a relatively intense high energy emission, while it is very weak for the bromide (**2**) and iodide (**3**) compounds (for details see Figure S19). We observe photochemical transformations occurring on the minute timescale, which lead to an intensity decrease of the high energy bands and an increase of the low energy emission. The latter shows pronounced shoulders in particular for **1** and **2**, suggesting the formation of multiple products. Interestingly, the relative rates of photoconversion increase along the series **1**<**2** ≪**3** and appear to be related to some extent to the Zn−X bond strengths (Figure 5B). We were able to determine a luminescence efficiency of the slowest transforming compound **1** of *ϕ*=0.03.

**Figure 6:**
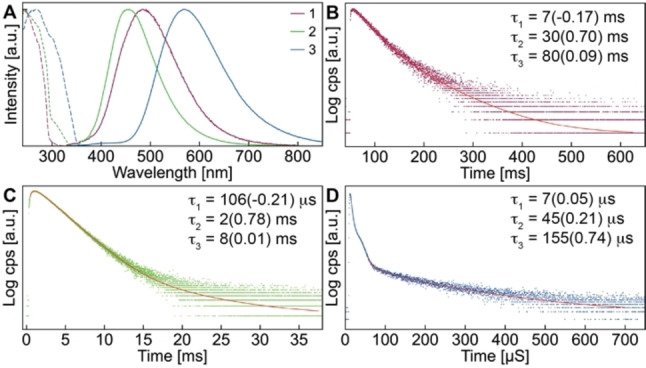
Emission and excitation spectra of dimers **1**–**3** (**A**) and their time‐resolved luminescence decays recorded at the respective *λ*_max_ (**C–D**) in the solid‐state at 77 K, red curves represent numerical fits (χ^2^ = 1.07 (**1**), 1.17 (**2**), 1.16 (**3**)) of the decay.

In an attempt to minimize the photochemical transformation, we studied the luminescence properties of **1**–**3** at 77 K (Figure 6A) and found that the steady‐state emission indeed does not change over time. Albeit very weak, high‐energy bands resembling the ones at 297 K are observed besides broad low energy bands that are – presumably due to reduced non‐radiative decay – much more intense and defined than at room temperature. The luminescence lifetimes recorded at the respective maxima of the low energy bands are bi‐ or tri‐exponential in the micro‐ to millisecond regime and decrease with increasing size of the halides (Figure 6B–D and Tables S4–S6). We therefore attribute the emission to stem from triplet excited states with weak SOC. Importantly, the time‐resolved measurements reveal rise‐times of 7 ms and 106 μs for **1** and **2**, respectively, which can be interpreted as the timescale at which the photo‐induced process occurs to reach the final emitting state. The relatively short lifetime of **3** does not allow to determine the rise‐time with our excitation lamp set‐up.

In order to identify the resulting new emissive species, we irradiated solid state samples at RT and at 77 K, and also solution samples of **1**–**3** overnight and tried to characterize the photoproducts by NMR spectroscopy or recrystallization from MeCN/Et_2_O solutions. However, in solution the original dimers were reformed, and thus the photochemical process is apparently reversible and the result of structural changes in the excited state. Irradiation of single crystals for direct characterization on the diffractometer did not produce meaningful data.

**Figure 7:**
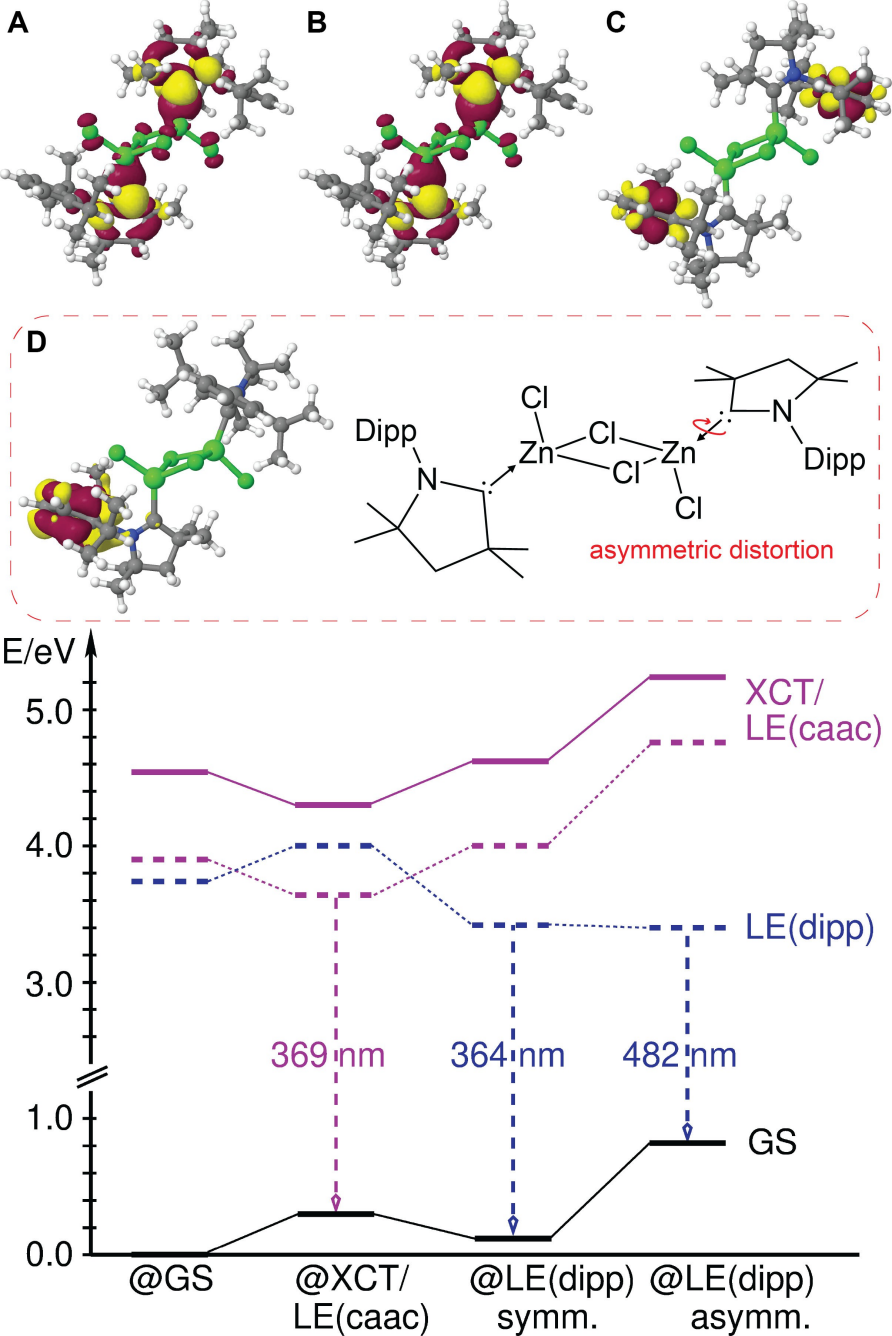
Energetic scheme and electron density differences of **1** in the (**A**) ^1^A_u_ XCT/LE (S_1u_) and (**B**) ^3^A_u_ XCT/LE states and (**C**) the C_i_‐symmetric and (**D**) localized asymmetric ^3^LE(ππ*_dipp_) states with regard to the S_0_ state at the relaxed excited‐state geometries. For color codes, see Figure 4.

The fact that the aforementioned high‐energy emission band of **1**–**3** is observed at the earliest stage of the luminescence measurements and its intensity continuously decreases under UV light irradiation, suggests that it belongs to the original, symmetrical dimeric arrangement. This hypothesis is further supported by quantum chemical studies. Our DFT/MRSOCI calculations confirm that the high‐energy band is phosphorescence in nature and originates from a ^3^A_u_ XCT/LE_cAAC_ state, which is the triplet counterpart of the S_1_ state and retains the inversion symmetry of the ground state (Figure 7A/B and Table S8).

The increasing degree of halide contribution and resulting operative SOC for the heavier congeners are reflected in shorter radiative lifetimes (*τ*=10 ms (**1**), 640 μs (**2**), 27 μs (**3**)) and more pronounced bathochromic shift of the luminescence wavelengths (*λ*_em_=369 (**1**), 372 (**2**) and 393 (**3**) nm), which is in good agreement with the experimental observations.

For compounds **1** and **2**, we were able to identify a photo‐induced excited state transformation that might be responsible for the broad low energy band observed in the experimental luminescence spectra. The ^3^LE(ππ*_dipp_) state forms a symmetry‐broken structure on the T_1_ potential energy surface with nearly perpendicular carbene orientations. Upon torsion of one cAAC ligand around the Zn−C bond, the excitation localizes on the other cAAC (Figure 7D). Its vertical emission wavelength of *λ*_em_=482 and 469 nm for **1** and **2**, respectively, coincides nicely with the experimental emission spectra at 77 K and the early spectra at room temperature, although for **2** this ^3^LE state appears as a shoulder (Figure 5) at 297 K besides other products (see below). The large conformational change in conjunction with the small stabilization energy of 0.04 eV with regard to the C_i_‐symmetric initial ^3^LE(ππ*_dipp_) structure explains the retarded build‐up of the lower‐energy band in the solid‐state and its enormous width. Due to its local ^3^ππ*_dipp_ character without significant chlorine or zinc contributions, the phosphorescence emission of this state is very long‐lived. The trend of the calculated radiative lifetimes of *τ*=3 s (**1**) and 79 ms (**2**) agrees with our findings (see above), although the observed lifetimes are shorter due to significant non‐radiative decay, as exemplified by *ϕ*_77K_=0.13 for **1**. For a ^3^LC state, one would not expect significant premature relaxation at such low temperatures. However, the relatively large distortion in comparison to the optimized geometry of the electronic ground state apparently provides low energy pathways via conical intersections.

We note that population of the localized asymmetric ^3^LE(ππ*_dipp_) state cannot explain the highly bathochromically shifted low energy band of **3**, leading us to conclude that other additional photochemical distortions occur that yet need to be identified. This interpretation would also explain the much stronger halide‐dependent kinetics of **3** depicted in Figure 5, and the multiple shoulders of the broad emission upon prolonged irradiation at room temperature for **1** and **2**.

**Figure 8:**
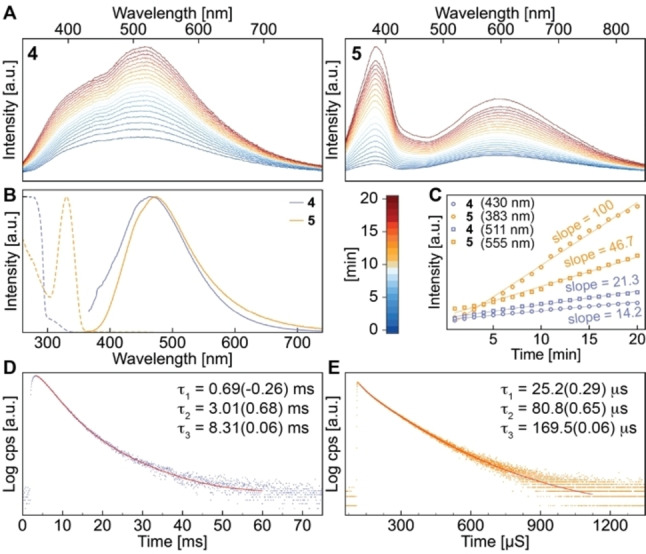
Emission and excitation spectra of monomers **4** and **5** in the solid‐state at 297 (**A**) and 77 K (**B**), emission intensity‐time plot (**C**) and luminescence lifetime decays of **4** (**D**) and **5** (**E**) at 77 K, red curves represent numerical fits (χ^2^ = 1.12 (**4**), 1.13 (**5**)).

The monomers [ZnX_2_(^Me^cAAC)(NCCH_3_)] (X=Br (**4**), I (**5**)) also undergo structural changes in the solid state under UV‐irradiation at room temperature (Figure 8A). In contrast to their dimeric congeners **2** and **3**, both high and low energy bands increase in intensity within minutes. At 77 K, the steady‐state luminescence does not undergo any changes and broad emission bands with *λ*_max_=470 (**4**) and 480 (**5**) nm are observed, and the time‐resolved measurements reveal decay times in the milli‐ and microsecond regime for **4** and **5**, respectively. It is noteworthy that the emission lifetime of **4** is very similar as found for the dimer **2**, and that again a rise‐time of 687 μs indicates a significant excited state distortion. Bearing in mind that the calculated local ^3^ππ*_dipp_ of all dimers is halide‐independent with regard to the emission wavelength of ca. 470 nm, but that the calculated decay times do decrease with the heavier analogues (Table S8), it is quite likely that for **4** and **5** the excited state transformation also yields a ^3^LE_dipp_ state at 77 K. At room temperature, more diverse photochemical changes can occur, which may include halide or solvent dissociation and even changes of intermolecular interactions.

## Conclusions

In this work, we have reported the synthesis and photophysical properties of a series of zinc(II) cyclic alkyl(amino)carbene complexes comprising both dimeric structures with bridging halide ligands, namely [(^Me^cAAC)XZn(μ‐X)_2_ZnX(^Me^cAAC)] (X=Cl (**1**), Br (**2**), I (**3**)), and monomeric species [ZnX_2_(^Me^cAAC)(NCCH_3_)] (X=Br (**4**), I (**5**)) containing coordinated acetonitrile. The bromido and iodo compounds appear to have a tendency to switch between the monomeric and dimeric forms depending on the reaction conditions, i.e., solvent and concentration.

All compounds show visible photoluminescence from triplet excited states with ultra‐long lifetimes (up to ms region), where the cAAC acts as π‐chromophore ligand. At room temperature, photochemical transformations in the excited state occur, leading to multiple emitting species, which are reversed into the original structures upon dissolution and recrystallization. However, at 77 K the transformation appears to be very specific, allowing to elucidate the reaction path by combined experimental and theoretical studies.

According to our DFT/MRCI calculations, the ^3^XCT/LE_caac_ excitations represent minima on the T_1u_ potential energy surface of the dimers, giving high‐energy phosphorescence with lifetimes in the milli‐ to microsecond range. However, the global minima on the T_1_ potential energy surfaces result from localized π_dipp_→π*_dipp_ excitations of the cAAC ligand, yielding a pair of triplet states which are located adiabatically slightly below the A_g_‐ and A_u_‐symmetric ^3^XCT/LE_caac_ states. The localization of the ^3^LE(ππ*_dipp_) excitation is accompanied by a large conformational change of the nuclear framework, i.e. rotation of the cAAC, but maintains its dimeric structure. While the relaxation energy to reach that minimum is small in the triplet state, the asymmetric distortion is very unfavourable in the electronic ground state, thus leading to a strong redshift of the long‐lived phosphorescence emission. The monomeric complexes **4** and **5** also appear to emit from ^3^LE(ππ*_dipp_) states at 77 K, but undergo more diverse transformations at room temperature.

Apart from porphyrin‐based systems, phosphorescent Zn^II^ complexes are extraordinarily rare. Bearing in mind that the ultra‐long luminescence lifetimes of the monomeric and dimeric species are mainly the result of the {(^Me^cAAC)Zn} acting as chromophore unit, modification of the coordination sphere should provide opportunities for further improvement of the photophysical properties. Also, the identification of the mechanism of photo‐induced transformation suggests potential routes for enhanced photostability, for example, halide exchange or increased steric demand of the ligand sphere. Our study thus represents an important step towards the design of new photoactive 3d metal complexes, and we will report on the mentioned modifications and their effects on the photophysics and photoreactivity of Zn^II^ carbene compounds shortly.

## Experimental Section

### Materials and Techniques

All operations were performed under an argon atmosphere by using conventional Schlenk‐line techniques or glovebox. The solvents were dried using Technology Inc. Pure‐Solv system or standard methods^[41]^ and degassed prior to use. The ^Me^cAAC was prepared according to a procedure reported in the literature.^[14]^ Zinc halides were dried by heating (up to 100 °C) under high vacuum. All other starting materials were available commercially and were used without further purification. ^1^H, ^13^C{^1^H} APT, ^1^H‐^13^C HSQC, ^1^H‐^13^C HSMB, ^1^H‐^1^H COSY and ^1^H‐^15^N HMBC were measured at 300 K on Bruker 500 Avance or Bruker 600 Avance spectrometers. The chemical shifts are given in ppm relative to residual signals of the solvent [^1^H, ^13^C: CD_3_CN (1.94, 118.69 ppm)]. MALDI‐HRMS MS spectra were recorded on a Thermo Scientific Q Exactive in positive ion mode connected with a TransMIT AP‐SMALDI containing a laser with a wavelength of 343 nm. Best results were achieved with an attenuator of 35°.

### Synthesis and Characterization

#### [(^Me^cAAC)ClZn(μ‐Cl)_2_ZnCl(^Me^cAAC)] (1)

Solutions of 100 mg (734 μmol) of ZnCl_2_ and 220 mg (770 μmol) of ^Me^cAAC each in 5 mL of THF were cooled to −40 °C, mixed and stirred 30 minutes at low temperature. After that, the mixture was allowed to slowly warm to room temperature over a period of 2 h, during which single‐crystals suitable for X‐ray diffraction analysis of **1** were formed. The supernatant was removed and the crystals were washed twice with 2 mL of THF and once with 2 mL of *n‐*pentane, and then vacuum dried to give white crystalline **1**. Yield: 260 mg (308 μmol, 84 %). Elemental analysis (%) calcd for C_40_H_62_N_2_Zn_2_Cl_4_: C, 56.95; H, 7.41; N, 3.31. Found: C, 57.0; H, 7.6; N, 3.4. ^1^H NMR (600 MHz, CD_3_CN): δ [ppm]=7.47 (m, 2H, H^4^, C_6_ *H*_3_^i^Pr_2_), 7.38 (m, 4H, H^3,5^, C_6_ *H*_3_^i^Pr_2_), 2.83 (sept, ^3^ *J*(^1^H,1H)=6.7 Hz, 4H, C*H* ^iPr^), 2.08 (s, 4H, −C*H*_2_−), 1.58 (s, 12H, (C*H*_3_)_2_), 1.40 (s, 12H, (C*H*_3_)_2_), 1.30 (d, ^3^ *J*(^1^H,1H)=6.7 Hz, 24H, C*H*_3_^iPr^). ^13^C NMR (151 MHz, CD_3_CN): δ [ppm]=247.9 (s, 2 C, *C* ^carbene^), 146.7 (s, 4 C, C^2,6^, Dipp), 135.0 (s, 2 C, C^1^, Dipp), 131.1 (s, 2 C, C^4^, Dipp), 126.2 (s, 4 C, C^3,5^, Dipp), 84.2 (s, 2 C, N*C*(CH_3_)_2_), 56.8 (s, 2 C, *C*(CH_3_)_2_C^carbene^), 50.1 (s, 2 C, −*C*H_2_−), 29.6 (s, 4 C, *C*H, Dipp), 29.4 (s, 4 C, C(*C*H_3_)_2_C^carbene^), 28.7 (s, 4 C, NC(*C*H_3_)_2_), 26.6 (s, 4 C, *C*H_3_, Dipp), 24.4 (s, 4 C, *C*H_3_, Dipp). ^15^N (61 MHz, CD_3_CN): δ [ppm]=−145.9.

#### [(^Me^cAAC)BrZn(μ‐Br)_2_ZnBr(^Me^cAAC)] (2)

Solutions of 165 mg (734 μmol) of ZnBr_2_ and 220 mg (770 μmol) of ^Me^cAAC, each in 2 mL of THF, were mixed and stirred at −40 °C for two hours. After that, crystallization was induced by slow diffusion of *n*‐pentane as antisolvent leading to single‐crystals suitable for X‐ray diffraction analysis. Precipitated product was collected, washed with 3 mL of *n*‐pentane and vacuum dried to afford white crystals of **2**. Yield: 285 mg (279 μmol, 76 %). Elemental analysis (%) calcd for C_40_H_62_N_2_Zn_2_Br_4_: C, 47.04; H, 6.12; N, 2.74. Found: C, 46.9; H, 6.0; N, 2.8. ^1^H NMR (600 MHz, CD_3_CN): δ [ppm]=7.48 (t, ^3^J(^1^H,^1^H)=7.8 Hz, 2H, H^4^, C_6_ *H*_3_^i^Pr_2_), 7.39 (d, ^3^J(^1^H,^1^H)=7.8 Hz, 4H, H^3,5^, C_6_ *H*_3_^i^Pr_2_), 2.83 (sept, ^3^ *J*(^1^H,1H)=6.7 Hz, 4H, C*H* ^iPr^), 2.08 (s, 4H, −CH_2_−), 1.65 (s, 12H, (C*H*_3_)_2_), 1.40 (s, 12H, (C*H*_3_)_2_), 1.34 (d, ^3^ *J*(^1^H,1H)=6.7 Hz, 12H, C*H*_3_^iPr^), 1.30 (d, ^3^ *J*(^1^H,1H)=6.7 Hz, 12H, C*H*_3_^iPr^). ^13^C NMR (151 MHz, CD_3_CN): δ [ppm]=246.8 (s, 2 C, *C* ^carbene^), 146.8 (s, 4 C, C^2,6^, Dipp), 134.9 (s, 2 C, C^1^, Dipp), 131.2 (s, 2 C, C^4^, Dipp), 126.3 (s, 4 C, C^3,5^, Dipp), 84.1 (s, 2 C, N*C*(CH_3_)_2_), 57.0 (s, 2 C, *C*(CH_3_)_2_C^carbene^), 50.4 (s, 2 C, −*C*H_2_−), 29.5 (m, 4, *C*H, Dipp, 4 C, C(*C*H_3_)_2_C^carbene^), 29.3 (s, 4 C, NC(*C*H_3_)_2_), 27.2 (s, 4 C, *C*H_3_, Dipp), 24.5 (s, 4 C, *C*H_3_, Dipp). ^15^N (61 MHz, CD_3_CN): δ [ppm]=‐146.0 ppm.

#### [(^Me^cAAC)IZn(μ‐I)_2_ZnI(^Me^cAAC)] (3)

Compound **3** was prepared in similar manner as described for **2** but with 234 mg (734 μmol) of ZnI_2_. Yield: 351 mg (290 μmol, 79 %). Elemental analysis (%) calcd for C_40_H_62_N_2_Zn_2_I_4_: C, 39.73; H, 5.17; N, 2.32. Found: C, 40.3; H, 5.2; N, 2.4. ^1^H NMR (600 MHz, CD_3_CN): δ [ppm]=7.51 (t, ^3^J(^1^H,^1^H)=7.7 Hz, 2H, H^4^, C_6_ *H*_3_^i^Pr_2_), 7.42 (d, ^3^J(^1^H,^1^H)=7.7 Hz, 4H, H^3,5^, C_6_ *H*_3_^i^Pr_2_), 2.87 (sept, ^3^ *J*(^1^H,1H)=6.6 Hz, 4H, C*H* ^iPr^), 2.10 (s, 4H, ‐CH_2_‐), 1.77 (s, 12H, (C*H*_3_)_2_), 1.40 (s, 12H, (C*H*_3_)_2_), 1.38 (d, ^3^ *J*(^1^H,1H)=6.6 Hz, 12H, C*H*_3_^iPr^), 1.31 (d, ^3^ *J*(^1^H,1H)=6.6 Hz, 12H, C*H*_3_^iPr^). ^13^C NMR (125 MHz, CD_3_CN): δ [ppm]=244.3 (s, 2 C, *C* ^carbene^), 146.9 (s, 4 C, C^2,6^, Dipp), 134.7 (s, 2 C, C^1^, Dipp), 131.2 (s, 2 C, C^4^, Dipp), 126.3 (s, 4 C, C^3,5^, Dipp), 83.8 (s, 2 C, N*C*(CH_3_)_2_), 57.7 (s, 2 C, *C*(CH_3_)_2_C^carbene^), 50.7 (s, 2 C, ‐*C*H_2_‐), 31.0 (s, 4 C, *C*H_3_), 29.3 (m, 4 C, *C*(CH_3_)_2_, 8 C, *C*H_3_), 24.6 (s, 4 C, *C*H_3_). ^15^N (60 MHz, CD_3_CN): δ [ppm]=−146.1 ppm.

#### [ZnBr_2_(^Me^cAAC)(NCCH_3_)] (4)

100 mg (98 μmol) of **2** was dissolved in 2 mL of CH_3_CN and the mixture was stirred at room temperature for one hour. After that, the solution was concentrated by vacuum evaporation of solvent to a volume of 0.5 mL and crystallization was induced by slow diffusion of Et_2_O as antisolvent. The product was collected, washed with 3 mL of Et_2_O and vacuum‐dried to afford colourless crystals of **4**. Yield: 94 mg (170 μmol, 87 %). Elemental analysis (%) calcd for C_22_H_34_N_2_ZnBr_2_: C, 47.90; H, 6.21; N, 5.08. Found: C, 48.1; H, 6.3; N, 5.2. ^1^H NMR (600 MHz, CD_3_CN): δ [ppm]=7.48 (t, ^3^J(^1^H,^1^H)=7.8 Hz, 1H, H^4^, C_6_ *H*_3_^i^Pr_2_), 7.39 (d, ^3^J(^1^H,^1^H)=7.8 Hz, 2H, H^3,5^, C_6_ *H*_3_^i^Pr_2_), 2.83 (sept, ^3^ *J*(^1^H,1H)=6.7 Hz, 2H, C*H* ^iPr^), 2.08 (s, 2H, ‐CH_2_‐), 1.94 (s, 3H, C*H*_3_CN), 1.65 (s, 6H, (C*H*_3_)_2_), 1.40 (s, 6H, (C*H*_3_)_2_), 1.34 (d, ^3^ *J*(^1^H,1H)=6.7 Hz, 6H, C*H*_3_^iPr^), 1.30 (d, ^3^ *J*(^1^H,1H)=6.7 Hz, 6H, C*H*_3_^iPr^). ^13^C NMR (125 MHz, CD_3_CN): δ [ppm]=246.8 (s, 1 C, *C* ^carbene^), 146.8 (s, 2 C, C^2,6^, Dipp), 134.9 (s, 1 C, C^1^, Dipp), 131.2 (s, 1 C, C^4^, Dipp), 126.3 (s, 2 C, C^3,5^, Dipp), 84.1 (s, 1 C, N*C*(CH_3_)_2_), 57.0 (s, 1 C, *C*(CH_3_)_2_C^carbene^), 50.4 (s, 1 C, ‐*C*H_2_‐), 29.5 (m, 2 C, *C*H, Dipp, 2 C, C(*C*H_3_)_2_C^carbene^), 29.3 (s, 2 C, NC(*C*H_3_)_2_), 27.2 (s, 2 C, *C*H_3_, Dipp), 24.5 (s, 2 C, *C*H_3_, Dipp). ^15^N (60 MHz, CD_3_CN): δ [ppm]=−146.0 ppm. Single crystals suitable for X‐ray diffraction analysis were prepared by slow diffusion of Et_2_O into solution of **4** in CH_3_CN.

#### [ZnI_2_(^Me^cAAC)(NCCH_3_)] (5)

Compound **5** was prepared in similar manner as described for **4** but with 100 mg (83 μmol) of **3**. Yield: 90 mg (139 μmol, 84 %). Elemental analysis (%) calcd for C_22_H_34_N_2_ZnI_2_: C, 40.92; H, 5.31; N, 4.34. Found: C, 40.9; H, 5.3; N, 4.6. ^1^H NMR (600 MHz, CD_3_CN): δ [ppm]=7.51 (t, ^3^J(^1^H,^1^H)=7.7 Hz, 1H, H^4^, C_6_ *H*_3_^i^Pr_2_), 7.42 (d, ^3^J(^1^H,^1^H)=7.7 Hz, 2H, H^3,5^, C_6_ *H*_3_^i^Pr_2_), 2.87 (sept, ^3^ *J*(^1^H,1H)=6.6 Hz, 2H, C*H* ^iPr^), 2.10 (s, 2H, ‐CH_2_‐), 1.96 (s, 3H, C*H*_3_CN), 1.77 (s, 6H, (C*H*_3_)_2_), 1.40 (s, 6H, (C*H*_3_)_2_), 1.38 (d, ^3^ *J*(^1^H,1H)=6.6 Hz, 6H, C*H*_3_^iPr^), 1.31 (d, ^3^ *J*(^1^H,1H)=6.6 Hz, 6H, C*H*_3_^iPr^). ^13^C NMR (125 MHz, CD_3_CN): δ [ppm]=244.3 (s, 1 C, *C* ^carbene^), 146.9 (s, 2 C, C^2,6^, Dipp), 134.7 (s, 1 C, C^1^, Dipp), 131.2 (s, 1 C, C^4^, Dipp), 126.3 (s, 2 C, C^3,5^, Dipp), 83.8 (s, 1 C, N*C*(CH_3_)_2_), 57.7 (s, 1 C, *C*(CH_3_)_2_C^carbene^), 50.7 (s, 1 C, ‐*C*H_2_‐), 31.0 (s, 2 C, *C*H_3_), 29.3 (m, 2 C, *C*(CH_3_)_2_, 4 C, *C*H_3_), 24.6 (s, 2 C, *C*H_3_). ^15^N (60 MHz, CD_3_CN): δ [ppm]=−146.2. Single crystals suitable for X‐ray diffraction analysis were prepared by slow diffusion of Et_2_O into a solution of **5** in CH_3_CN.

#### ^Me^cAAC(H)CH_2_CN (6)

36.5 mg of ^Me^cAAC (129 μmol) was dissolved in 2 mL of CH_3_CN and the solution was stirred at room temperature for one hour. After that, the solvent was vacuum evaporated to give white powder of compound **6**. Yield: 43.5 mg (127 mg, 98.5 %). ^1^H NMR (600 MHz, CDCl_3_): 7.28 (m, 1H, H^4^, Dipp), 7.19 (m, 2H, H^3,5^, Dipp), 3.82 (dd, ^3^ *J*(^1^H,^1^H)=3.9 Hz, ^3^ *J*(^1^H,^1^H)=10.5 Hz, 1H, C(*H*)CH_2_CN), 3.72 (sept, ^3^ *J*(^1^H,^1^H)=6.8 Hz, 1H, C*H* ^iPr^), 3.27 (sept, ^3^ *J*(^1^H,^1^H)=6.8 Hz, 1H, C*H* ^iPr^), 2.22–2.10 (AB_2_ pattern, *J*_AB_=14.7 Hz, Δν/J_AB_=2.5, 2H, C*H*_2_CN), 2.01 (AB pattern, Δδ_AB_=0.1 ppm, ^2^ *J*(^1^H,^1^H)=13.1 Hz, −C*H*_2_−), 1.37 (s, 3H, CH(C*H*_3_)_2_CH_2_), 1.34 (s, 3H, CH(C*H*_3_)_2_CH_2_), 1.32 (d, ^3^ *J*(^1^H,^1^H)=6.8 Hz, 3H, CH(C*H*_3_)_2_), 1.25 (m, 6H, CH(C*H*_3_)_2_, 3H, NC(C*H*_3_)_2_CH_2_), 1.11 (d, ^3^ *J*(^1^H,^1^H)=6.8 Hz, 3H, CH(C*H*_3_)_2_), 1.06 (s, 3H, NC(C*H*_3_)_2_CH_2_). ^13^C NMR (125 MHz, CDCl_3_): δ [ppm]=152.2 (s, 1 C, C^2/6^, Dipp), 151.27 (s, 1H, C^2/6^, Dipp), 136.1 (s, 1 C, C^1^, Dipp), 127. 3 (s, 1 C, C^4^, Dipp), 125.0 (s, 1 C, C^3/5^, Dipp), 124.5 (s, 1 C, C^3/5^, Dipp), 118.6 (s, 1 C, CH_2_ *C*N), 69.1 (s, 1 C, N*C*HC(CH_3_)_2_), 61.4 (s, 1 C, CH*C*(CH_3_)_2_CH_2_), 57.0 (s, 1 C, C(CH_3_)_2_ *C*H_2_C(CH_3_)_2_), 39.5 (s, 1 C, N*C*(CH_3_)_2_CH_2_), 31.2 (s, 1 C, NC(*C*H_3_)_2_CH_2_), 29.6 (s, 1 C, CHC(*C*H_3_)_2_CH_2_), 29.2 (s, 1 C, *C*H(CH_3_)_2_), 28.9 (s, 1 C, NC(*C*H_3_)_2_CH_2_), 27.1 (s, 1 C, *C*H(CH_3_)_2_), 25.8 (s, 1 C, CH(*C*H_3_)_2_), 25.7 (s, 1 C, CH(*C*H_3_)_2_), 24.5 (s, 1 C, CH(*C*H_3_)_2_), 24.4 (s, 1 C, CHC(*C*H_3_)_2_CH_2_), 23.8 (s, 1 C, CH(*C*H_3_)_2_), 19.2 (s, 1 C, *C*H_2_CN). ^15^N (60 MHz, CDCl_3_): δ [ppm]=−133.8 (s, 1 N, C*N*), −316.9 (s, 1 N, CH*N*C(CH_3_)_2_). Single‐crystals suitable for X‐ray diffraction analysis were obtained from saturated solution of **6** in *n*‐pentane either by cooling to −40 °C or by slow evaporation.

Deposition Numbers 2161079 (for **1**), 2161084 (for **2**), 2161082 (for **3**), 2161081 (for **4**), 2161085 (for **5**), 2161083 (for **6**), 2161078 (for [^Me^cAACH]_2_[Zn_2_Cl_6_]), 2161080 (for [^Me^cAACH]_2_[ZnCl_4_]) contain the supplementary crystallographic data for this paper. These data are provided free of charge by the joint Cambridge Crystallographic Data Centre and Fachinformationszentrum Karlsruhe Access Structures service.

### Computational Studies

The quantum chemical calculations were performed using the Turbomole program package^[42]^ for all geometry optimizations and for the generation of the molecular orbitals and two‐electron integrals while the DFT/MRCI program[^43^, ^44^] and the spin−orbit coupling kit Spock^[45]^ were applied for computing the spectra and other photophysical properties of the complexes. Solvent effects were accounted for by the implicit solvent model COSMO[^46^, ^47^] using dielectric constants of ϵ=4.711 for chloroform and ϵ=37.50 for acetonitrile solutions. To model the adducts **4** and **5**, one CH_3_CN molecule was explicitly included in the quantum chemical treatment in addition to the COSMO surrounding. The equilibrium geometries of the electronic ground states were determined with Kohn−Sham density functional theory (DFT) employing the BH‐LYP[^48^, ^49^] functional including empirical dispersion corrections.[^50^, ^51^] Singlet excited‐state geometries of the isolated complexes were optimized at the level of time‐dependent DFT (TDDFT).^[52]^ Triplet‐state geometry optimizations additionally made use of the Tamm‐Dancoff approximation.^[53]^ Solvent effects on the nuclear arrangements of the complexes in the excited states were neglected. Carbon, nitrogen, chlorine and hydrogen atoms were represented by def‐SV(P) basis sets^[54]^ from the Turbomole basis set library. For the Zn ion, the relativistic small‐core effective core potential (ECP) defpp‐ECP and the associated contracted 6 s5p3d basis set were chosen.^[55]^ The heavier halogenides employed relativistic small‐core ECPs as well (ecp‐10‐mdf for bromine and ecp‐28‐mdf for iodine) in conjunction with the cc‐pVTZ‐PP basis for bromine and the SVP basis for iodine.^[56]^ Electronic excitation energies and oscillator strengths of the spin‐allowed transitions were calculated with the DFT/MRCI method employing the redesigned R2016 Hamiltonian.^[57]^ Due to the large size of the complexes, a tight configuration selection criterion (threshold 0.80 E_h_) was used. To compute the absorption spectra, the DFT/MRCI secular equations were solved for 20 singlet and 20 triplet roots, except for the C_i_‐symmetric dimers **2** and **3** where more states were required to cover the spectral range up to 200 nm. In addition to 20 ^1^A_g_ and ^3^A_g_ states, 20 (30) ^1^A_u_ and ^3^A_u_ states were computed for compound **2** (**3**), respectively. Because of the large spin‐orbit coupling (SOC) constants of the heavier halogenides, spectra were determined including SOC at the level of quasi‐degenerate perturbation theory using Spock.^[45]^ The Breit‐Pauli SOC integrals, employed for C, N, H and Cl, were evaluated in the atomic mean‐field approximation using AMFI, whereas spin‐orbit ECPs were used for Zn, Br and I.[^55^, ^56^, ^58^, ^59^, ^60^] To avoid convergence problems of the perturbation expansions, phosphorescence rate constants were determined using the multireference spin‐orbit configuration interaction (MRSOCI) approach implemented in Spock.^[61]^

### Photophysical Measurements

To avoid decomposition of samples and quenching of photoluminescence, all photophysical measurements were performed under an inert atmosphere of argon. The acetonitrile used for electronic spectra measurement was stored over activated 4 Å molecular sieves and purified (to remove traces of molecular sieves and oxygen) by vacuum‐transfer technique prior to use. Absorption spectra were recorded on a Cary 5000 UV‐Vis‐NIR spectrophotometer in the range 200–800 nm using quartz cuvettes with 1 cm path length. The concentration of samples was adjusted in the range of 1–7×10^−5^ mol/l. Emission and excitation spectra with lifetimes were recorded on an Edinburgh Instrument FLS1000 spectrometer, equipped with a double monochromator for the excitation and emission pathways and a red‐sensitive photomultiplier (PMT‐980, 200–880 nm) as detector. For measurements of emission and excitation spectra, 450 W Xenon arc lamp was used, whereas lifetimes were recorded using a pulsed 60 W Xenon microsecond flashlamp with a repetition rate of 1–100 Hz. Low temperature measurements were performed using an Oxford Optistat DN cryostate.

## Conflict of interest

The authors declare no conflict of interest.

1

## Supporting information

As a service to our authors and readers, this journal provides supporting information supplied by the authors. Such materials are peer reviewed and may be re‐organized for online delivery, but are not copy‐edited or typeset. Technical support issues arising from supporting information (other than missing files) should be addressed to the authors.

Supporting Information

## Data Availability

The data that support the findings of this study are available in the supplementary material of this article.
