# Supplementary material for: Ultra‐Long Lived Luminescent Triplet Excited States in Cyclic (Alkyl)(amino)carbene Complexes of Zn(II) Halides
Source: Chemistry. 2022 Jun 21;28(45):e202201114. doi: 10.1002/chem.202201114 (PMC9544448; doi:10.1002/chem.202201114)
Supplement: Supplementary file 1 — Supporting Information [file CHEM-28-0-s001.pdf]

Chemistry–A European Journal

Supporting Information

Ultra-Long Lived Luminescent Triplet Excited States in Cyclic (Alkyl)(amino)carbene Complexes of Zn(II) Halides

Ondřej Mrózek, Markus Gernert, Andrey Belyaev, Mousree Mitra, Lars Janiak, Christel M. Marian,* and Andreas Steffen*

1. NMR spectra 2

2. Crystallographic Details 6

3. Mass spectroscopy 11

4. Photophysical measurement 15

4.1. Absorption spectra 15

4.2. Measurements of photoinduced transformation in solid state 16

4.3. Low temperature measurement in solid state 20

4.3.1. Emission and excitations profiles 20

4.3.2. Details of lifetime measurements in solid state 23

5. Computational details 28

6. References .... *Fehler! Textmarke nicht definiert.*

1. NMR spectra

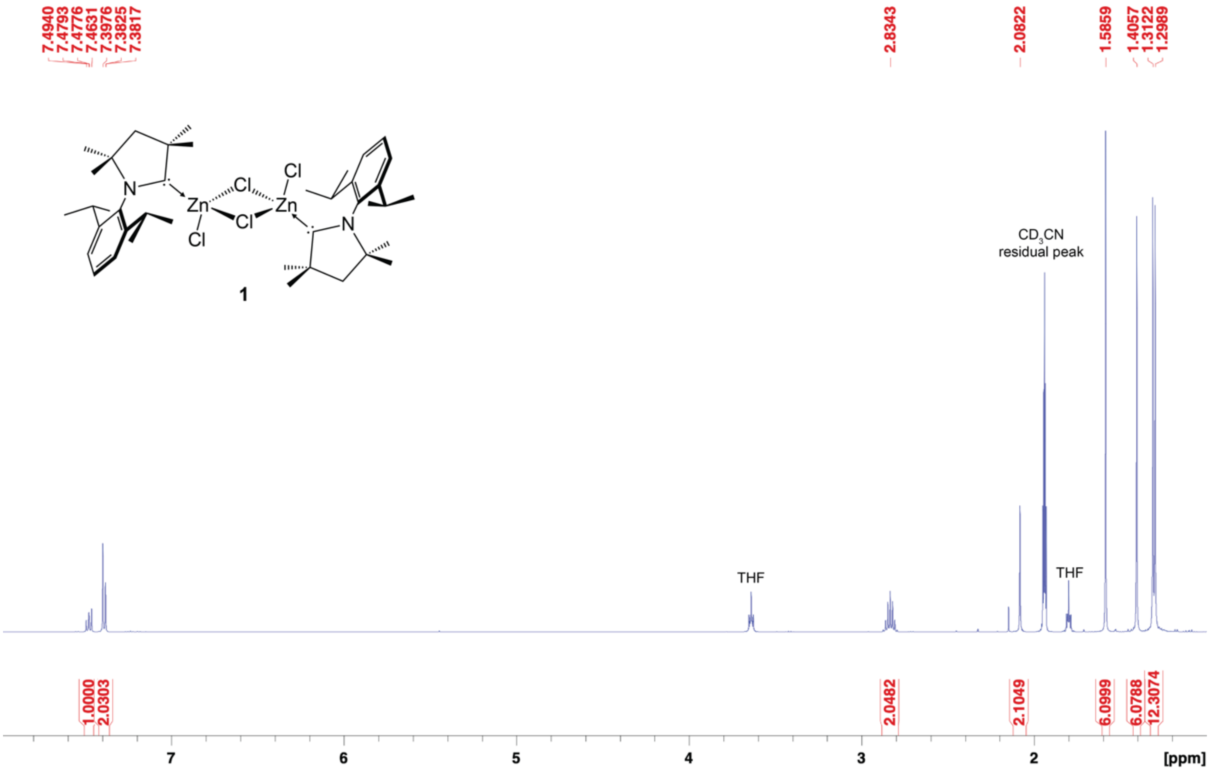

Figure S1 ^{1}H NMR spectrum of **1** in CD_3CN.

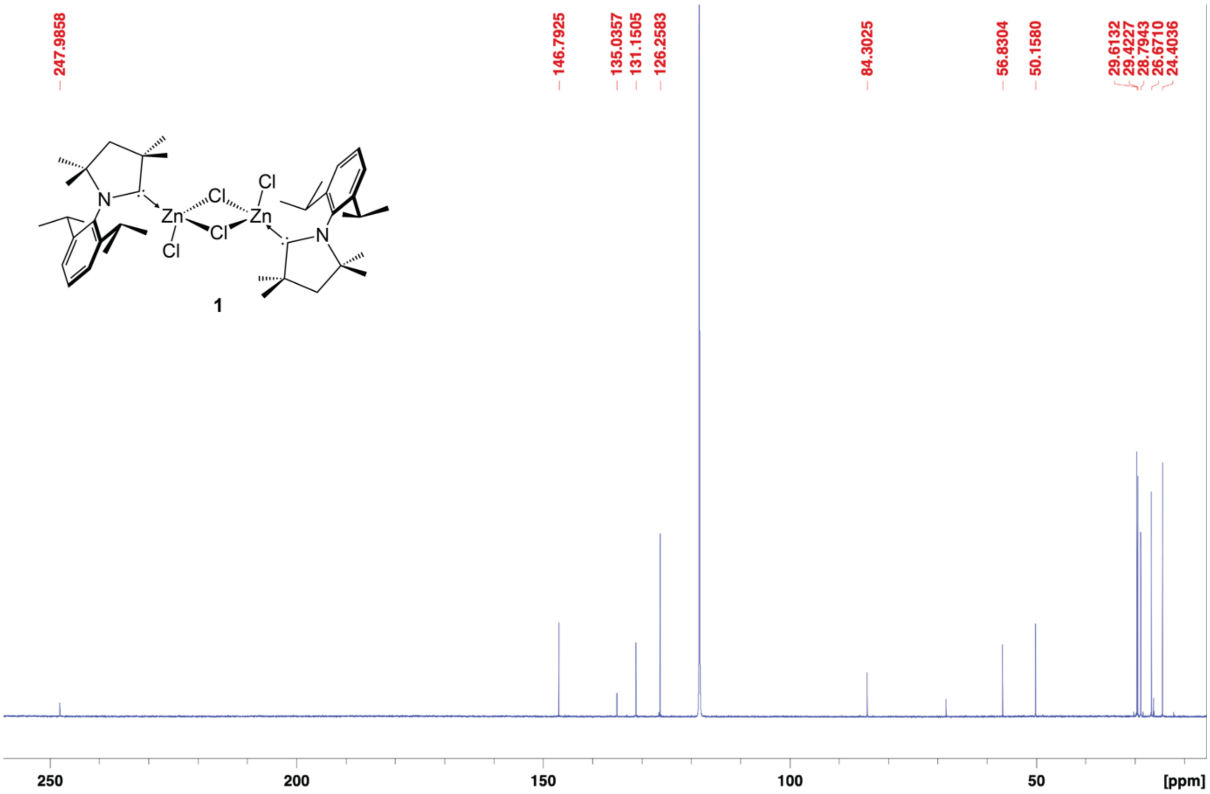

Figure S2 ^{13}C NMR spectrum of **1** in CD_3CN.

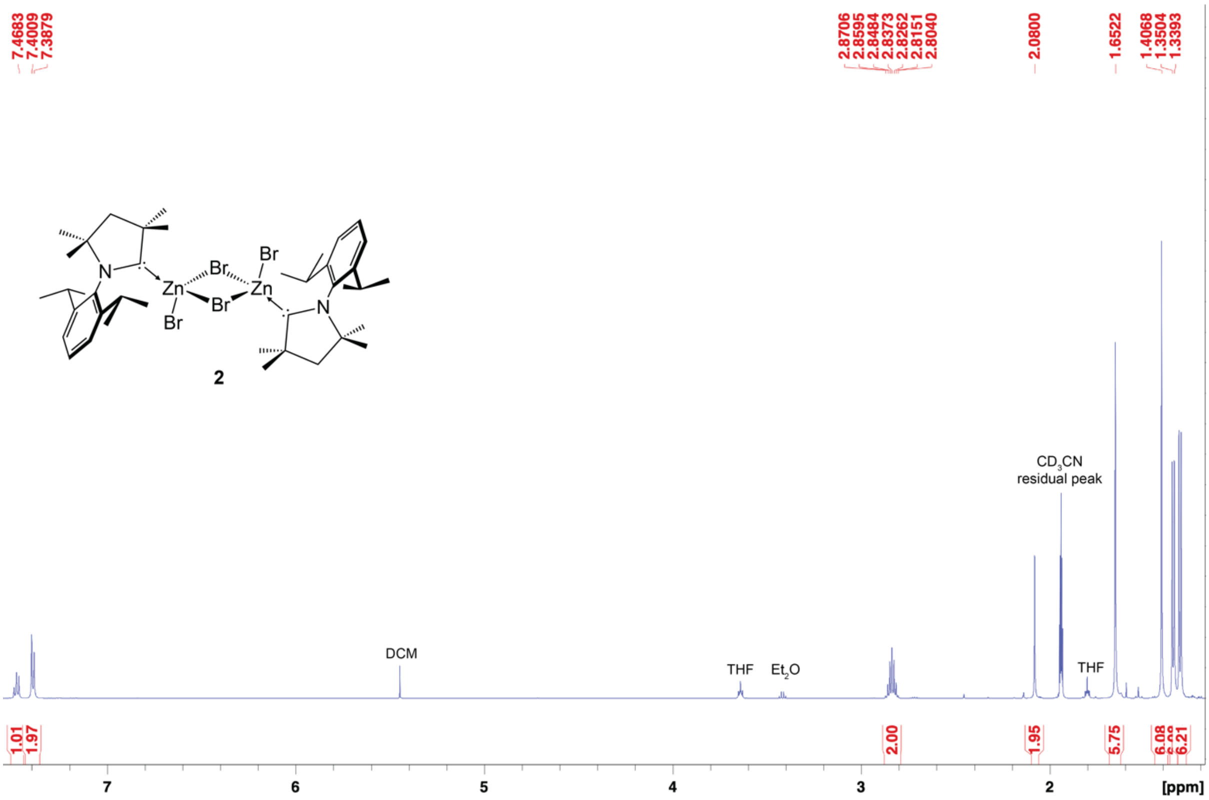

Figure S3 ^{1}H NMR spectrum of **2** in CD_3CN.

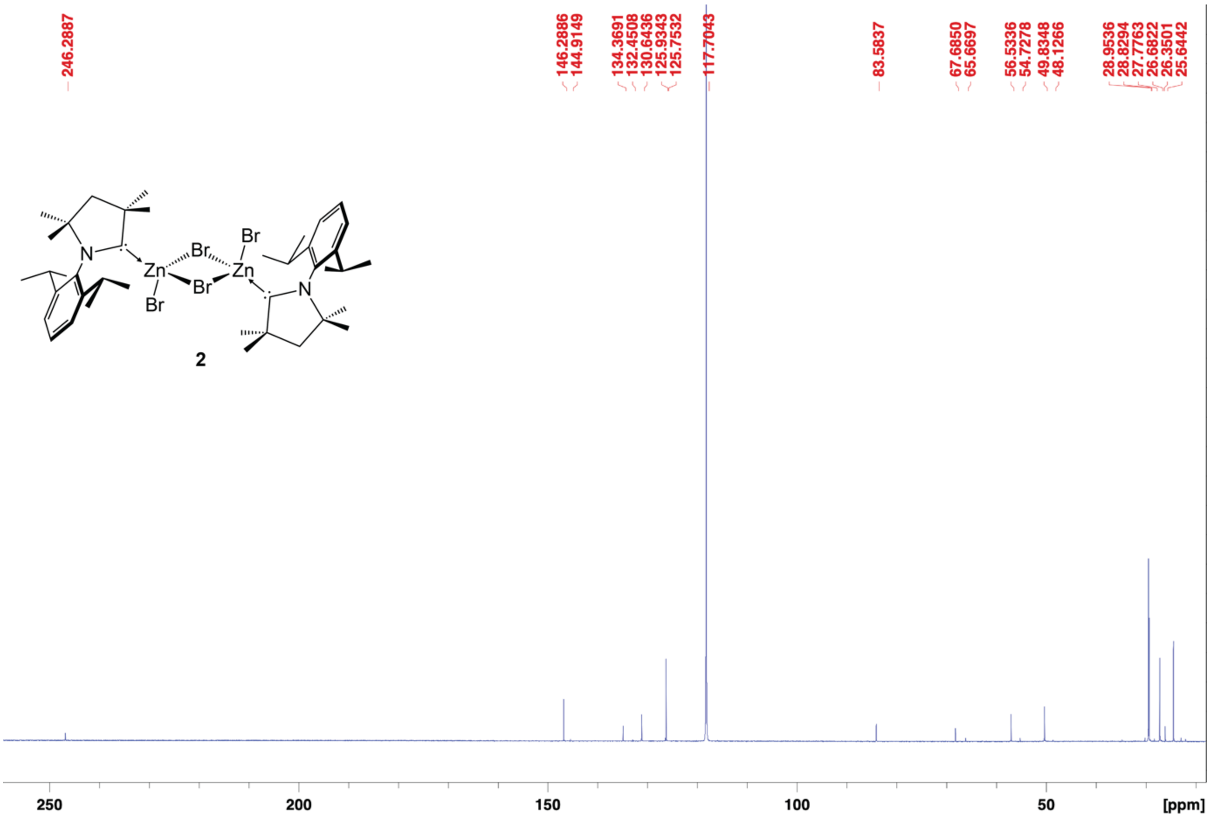

Figure S4 ^{13}C NMR spectrum of **2** in CD_3CN.

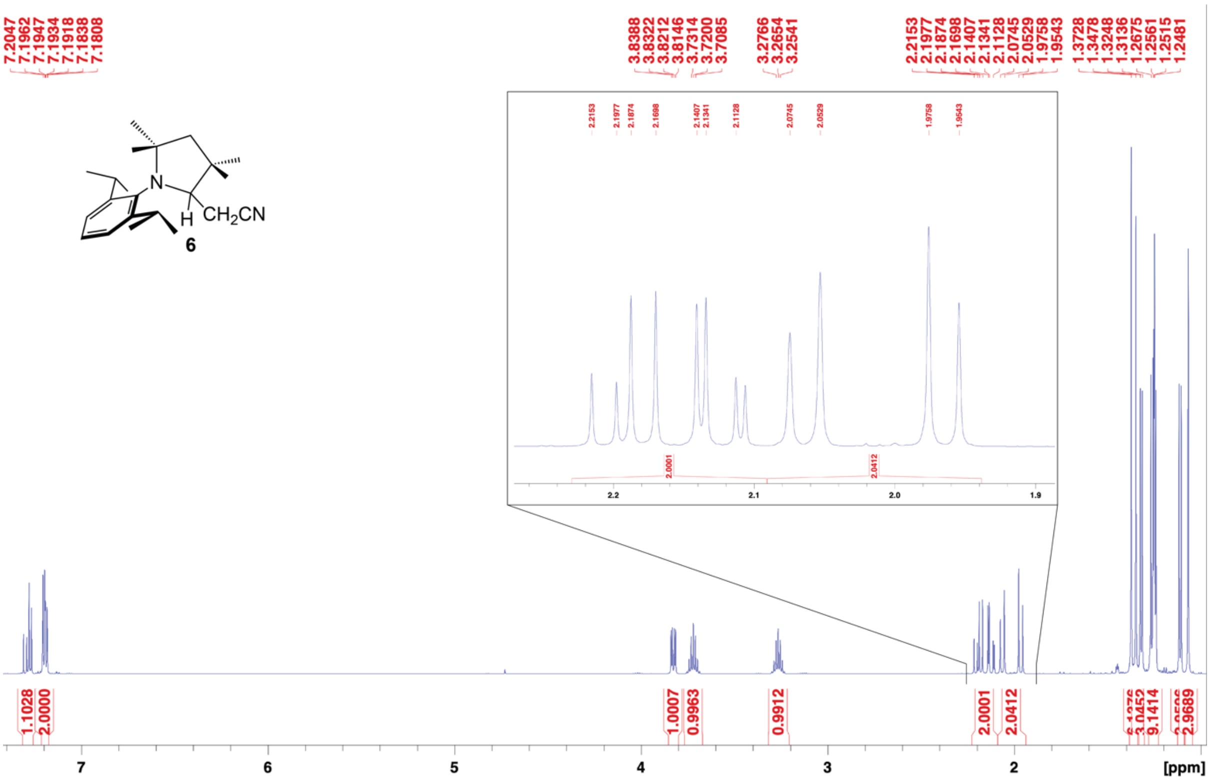

Figure S5 ^{1}H NMR spectrum of **6** in $CDCl_3$.

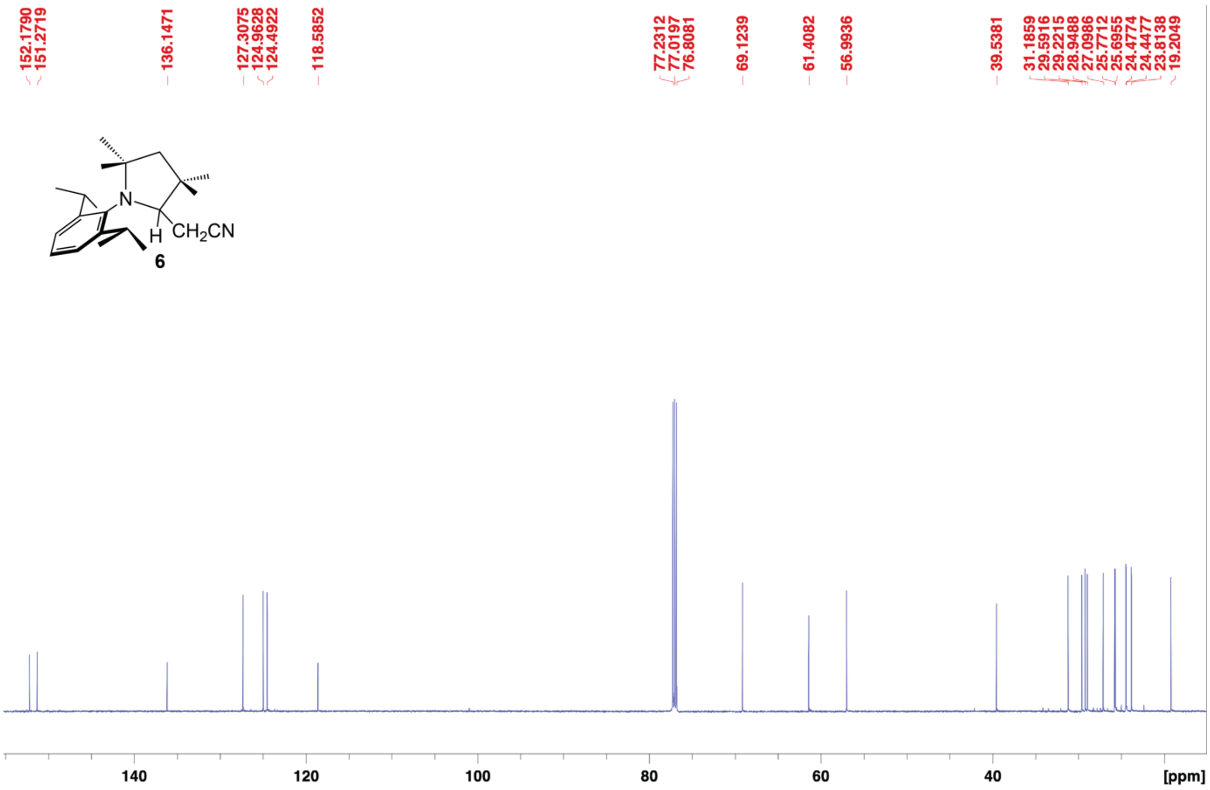

Figure S6 ^{13}C NMR spectrum of **6** in $CDCl_3$.

2. Crystallographic Details

The crystals of **1–6, $[CAACH]_2[Zn_2Cl_6]$** and **$[CAACH]_2[ZnCl_4]$** were immersed in a film of NVH or perfluoropolyether oil, mounted on a polyimide microloop (MicroMounts of MiTeGen) and transferred to stream of cold nitrogen (Bruker Kryoflex2), and measured at a temperature of 100–120 K. The X-ray diffraction data were collected on a Bruker D8 or Bruker X8/APEX II Venture diffractometers with a CMOS Photon 100 and multilayer optics monochromated MoKα (0.71073Å) radiation (INCOATEC microfocus sealed tube). The frames were integrated with the Bruker SAINT software package using a narrow-frame algorithm. The APEX2 v2014.9 and APEX3 v2018.7-0 program package was used for cell refinements and data reductions. The structure was solved using intrinsic phasing method,[1,2] refined and visualized with the OLEX2-1.3 program.[3] A semiempirical absorption correction (SADABS) was applied to all data. All non-hydrogen atoms were refined anisotropically. Hydrogen atoms were included in structure factors calculations. All Hydrogen atoms were assigned to idealised geometric positions. The unit cell of the **5** contains disordered solvent molecule of acetonitrile which has been treated as a diffuse contribution to the overall scattering without specific atom positions by SQUEEZE/PLATON.[4] The crystallographic details are summarized in Tables S1–S3. CCDC 2161078-2161085, contain the supplementary crystallographic data for this paper.

Table S1. Crystal data and structure refinement for **1-3**.

Identification code	**1**	**2**	**3**
CCDC number	**2161079**	**2161084**	**2161082**
Empirical formula	$C_{20}H_{31}Cl_2NZn$	$C_{20}H_{31}Br_2NZn$	$C_{20}H_{31}I_2NZn$
Formula weight	421.73	510.65	604.63
Temperature/K	120.0	120.0	100
Crystal system	monoclinic	monoclinic	monoclinic
Space group	$P2_1/n$	$P2_1/n$	$P2_1/n$
a/Å<br>b/Å<br>c/Å<br>α/°<br>β/°<br>γ/°	9.0591(2)<br>14.4133(3)<br>15.6058(3)<br>90<br>91.0780(10)<br>90	9.2453(2)<br>14.4651(3)<br>15.6827(3)<br>90<br>90.7760(10)<br>90	9.4595(4)<br>14.4196(7)<br>16.2337(8)<br>90<br>90.712(2)<br>90
Volume/$Å^3$	2037.31(7)	2097.12(7)	2214.14(18)
Z	4	4	4
$\rho_{calc}g/cm^3$	1.375	1.617	1.814
μ/mm^{-1}	1.470	4.984	3.900
F(000)	888.0	1032.0	1176.0
Crystal size/mm^3	0.1 × 0.08 × 0.06	0.11 × 0.09 × 0.06	0.1 × 0.08 × 0.07
Radiation	MoKα<br>(λ = 0.71073)	MoKα<br>(λ = 0.71073)	MoKα<br>(λ = 0.71073)
2Θ range for data collection/°	5.222 to 54.99	5.23 to 59.998	5.15 to 55
Index ranges	-11 ≤ h ≤ 11,<br>-18 ≤ k ≤ 18,<br>-20 ≤ l ≤ 20	-13 ≤ h ≤ 13,<br>-20 ≤ k ≤ 20,<br>-22 ≤ l ≤ 22	-12 ≤ h ≤ 11,<br>-18 ≤ k ≤ 18,<br>-21 ≤ l ≤ 21
Reflections collected	41228	103526	51254
Independent reflections	4673<br>[R_{int} = 0.0538,<br>R_{sigma} = 0.0269]	6093<br>[R_{int} = 0.0505,<br>R_{sigma} = 0.0186]	5088<br>[R_{int} = 0.0491,<br>R_{sigma} = 0.0234]
Data/restraints/parameters	4673/0/225	6093/0/225	5088/0/225
Final R indexes [I>=2σ (I)] [(a)]	R_1 = 0.0286,<br>wR_2 = 0.0669	R_1 = 0.0197,<br>wR_2 = 0.0428	R_1 = 0.0224,<br>wR_2 = 0.0540
Final R indexes [all data] [(a)]	R_1 = 0.0382,<br>wR_2 = 0.0711	R_1 = 0.0244,<br>wR_2 = 0.0443	R_1 = 0.0278,<br>wR_2 = 0.0558
Goodness-of-fit on F^2 [(b)]	1.071	1.049	1.053
Largest diff. peak/hole / e $Å^{-3}$	0.41/-0.34	0.52/-0.38	0.95/-0.84

[(a)] $R_1 = \sum||F_o|-|F_c||/\sum|F_o|$; $wR_2 = [\sum[w(F_o^2-F_c^2)^2/\sum[(wF_o^2)^2]]^{1/2}$; $w = 1/[\sigma^2(F_o^2)+(aP)^2+bP]$, where $P = (F_o^2+2F_c^2)/3$ [(b)] GooF = S = $[[\sum w(F_o^2-F_c^2)^2]/(m-n)]^{1/2}$, where m = number of reflexes and n = number of parameters

Table S2. Crystal data and structure refinement for **3-6**.

Identification code	**4**	**5**	**6**
CCDC number	**2161081**	**2161085**	**2161083**
Empirical formula	$C_{22}H_{34}Br_2N_2Zn$	$C_{22}H_{34}I_2N_2Zn$	$C_{22}H_{34}N_2$
Formula weight	551.70	645.68	326.51
Temperature/K	100.0	100.0	100.0
Crystal system	orthorhombic	orthorhombic	orthorhombic
Space group	Pbca	Pbca	Pbca
a/Å<br>b/Å<br>c/Å<br>α/°<br>β/°<br>γ/°	9.1653(3)<br>16.6534(5)<br>31.8098(7)<br>90<br>90<br>90	10.4806(6)<br>16.7116(9)<br>31.4704(16)<br>90<br>90<br>90	17.214(2)<br>10.5591(10)<br>22.109(3)<br>90<br>90<br>90
Volume/Å^3	4855.2(2)	5512.0(5)	4018.6(8)
Z	8	8	8
ρ_{calc}g/cm^3	1.509	1.556	1.079
μ/mm^{-1}	4.313	3.140	0.062
F(000)	2240.0	2528.0	1440.0
Crystal size/mm^3	0.12 × 0.098 × 0.052	0.24 × 0.218 × 0.145	0.11 × 0.08 × 0.05
Radiation	MoKα<br>(λ = 0.71073)	MoKα<br>(λ = 0.71073)	MoKα<br>(λ = 0.71073)
2Θ range for data collection/°	5.056 to 55	5.52 to 55	4.89 to 49.98
Index ranges	-11 ≤ h ≤ 10,<br>-18 ≤ k ≤ 21,<br>-35 ≤ l ≤ 41	-13 ≤ h ≤ 13,<br>-21 ≤ k ≤ 21,<br>-40 ≤ l ≤ 40	-20 ≤ h ≤ 20,<br>-12 ≤ k ≤ 12,<br>-25 ≤ l ≤ 26
Reflections collected	34347	125088	26007
Independent reflections	5562<br>[R_{int} = 0.0502,<br>R_{sigma} = 0.0381]	6325<br>[R_{int} = 0.0430,<br>R_{sigma} = 0.0163]	3537<br>[R_{int} = 0.1448,<br>R_{sigma} = 0.0779]
Data/restraints/parameters	5562/0/253	6325/0/254	3537/0/225
Final R indexes [I>=2σ (I)] [(a)]	R_1 = 0.0315,<br>wR_2 = 0.0602	R_1 = 0.0352,<br>wR_2 = 0.0885	R_1 = 0.0973,<br>wR_2 = 0.2390
Final R indexes [all data] [(a)]	R_1 = 0.0454,<br>wR_2 = 0.0642	R_1 = 0.0364,<br>wR_2 = 0.0891	R_1 = 0.1496,<br>wR_2 = 0.2721
Goodness-of-fit on F^2 [(b)]	1.029	1.212	1.079
Largest diff. peak/hole / e Å^{-3}	0.59/-0.38	1.73/-0.81	0.52/-0.30

[(a)] $R_1 = \sum||F_o|-|F_c||/\sum|F_o|$; $wR_2 = [\sum[w(F_o^2-F_c^2)^2/\sum[(wF_o^2)^2]]^{1/2}$; $w = 1/[\sigma^2(F_o^2)+(aP)^2+bP]$, where $P = (F_o^2+2F_c^2)/3$ [(b)] GooF = S = $[[\sum w(F_o^2-F_c^2)^2]/(m-n)]^{1/2}$, where m = number of reflexes and n = number of parameters

Table S3. Crystal data and structure refinement for **$[CAACH]_2[Zn_2Cl_6]$** and **$[CAACH]_2[ZnCl_4]$**.

Identification code	**[CAACH]2[Zn2Cl6]**	**[CAACH]2[ZnCl4]**
CCDC number	**2161078**	**2161080**
Empirical formula	$C_{20}H_{32}Cl_3NZn$	$C_{40}H_{64}Cl_4N_2Zn$
Formula weight	458.18	780.10
Temperature/K	100.15	100.15
Crystal system	monoclinic	triclinic
Space group	$P2_1/n$	P-1
a/Å<br>b/Å<br>c/Å<br>α/°<br>β/°<br>γ/°	9.494(5)<br>16.128(6)<br>14.726(8)<br>90<br>96.086(18)<br>90	10.086(7)<br>11.135(6)<br>19.690(9)<br>105.49(3)<br>98.65(3)<br>92.95(4)
Volume/$Å^3$	2242.2(19)	2097(2)
Z	4	2
$\rho_{calc}g/cm^3$	1.357	1.235
μ/mm^{-1}	1.456	0.869
F(000)	960.0	832.0
Crystal size/mm^3	0.561 × 0.524 × 0.32	0.3 × 0.217 × 0.11
Radiation	MoKα (λ = 0.71073)	MoKα (λ = 0.71073)
2Θ range for data collection/°	4.88 to 52.034	3.814 to 53.62
Index ranges	-11 ≤ h ≤ 11, -19 ≤ k ≤ 19, -18 ≤ l ≤ 18	-12 ≤ h ≤ 12, -11 ≤ k ≤ 14, -24 ≤ l ≤ 24
Reflections collected	20399	50524
Independent reflections	4415 [Rint = 0.0344, Rsigma = 0.0276]	8961 [R_{int} = 0.0434, R_{sigma} = 0.0363]
Data/restraints/parameters	4415/0/234	8961/0/440
Final R indexes [I>=2σ (I)] (a)	1.035	1.013
Final R indexes [all data] (a)	R1 = 0.0223, wR2 = 0.0500	R_1 = 0.0283, wR_2 = 0.0598
Goodness-of-fit on F^2 (b)	R1 = 0.0286, wR2 = 0.0524	R_1 = 0.0412, wR_2 = 0.0643
Largest diff. peak/hole / e $Å^{-3}$	0.38/-0.24	0.43/-0.33

(a) $R_1 = \sum||F_o|-|F_c||/\sum|F_o|$; $wR_2 = [\sum[w(F_o^2-F_c^2)^2/\sum[(wF_o^2)^2]]^{1/2}$; $w = 1/[\sigma^2(F_o^2)+(aP)^2+bP]$, where $P = (F_o^2+2F_c^2)/3$ (b) GooF = S = $[[\sum w(F_o^2-F_c^2)^2]/(m-n)]^{1/2}$, where m = number of reflexes and n = number of parameters

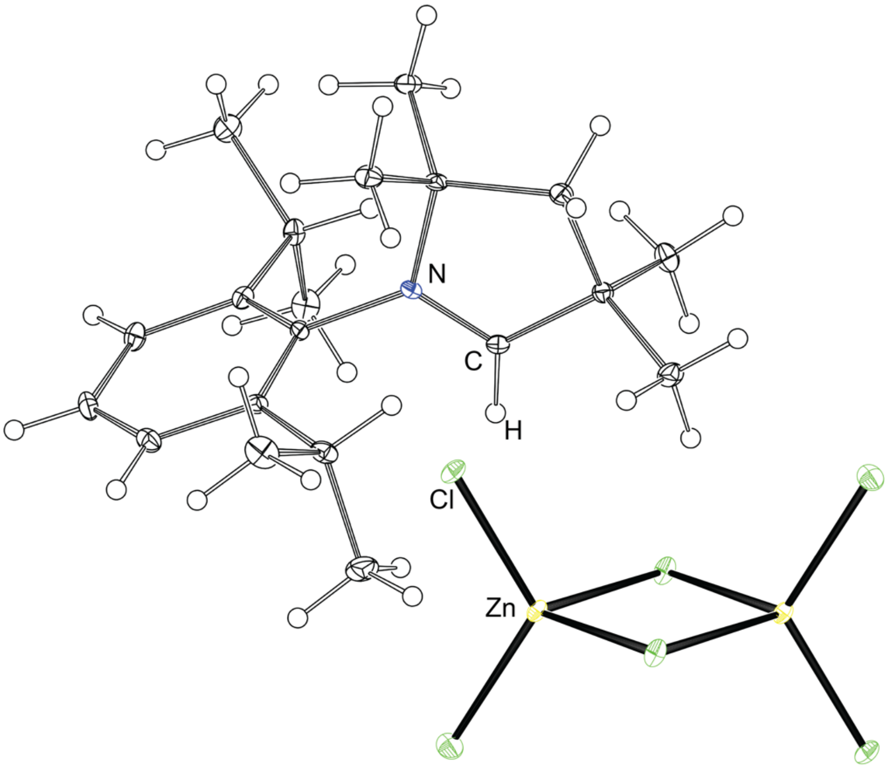

Figure S7 ORTEP view of side product **[CAACH]$_2$[Zn$_2$Cl$_6$].** Thermal ellipsoids are drawn at the 30% probability level.

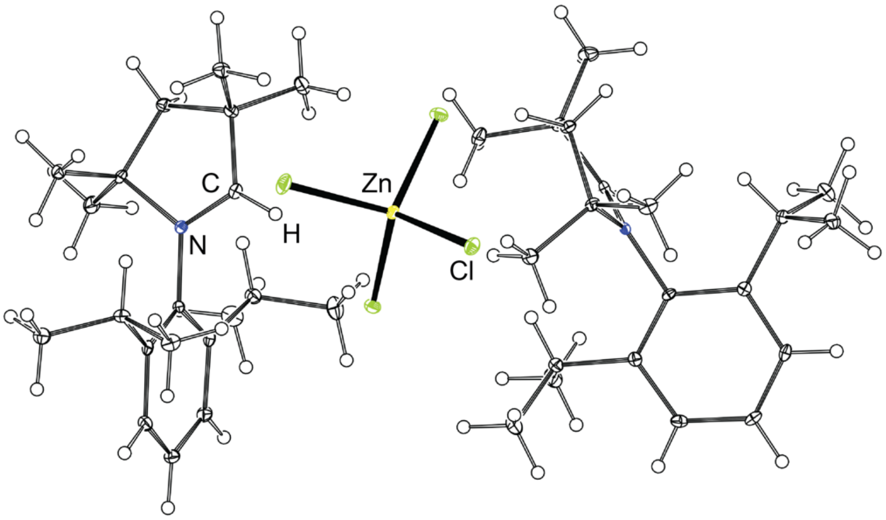

Figure S8 ORTEP view of side product **[CAACH]$_2$[ZnCl$_4$]**. Thermal ellipsoids are drawn at the 30% probability level.

3. Mass spectrometry

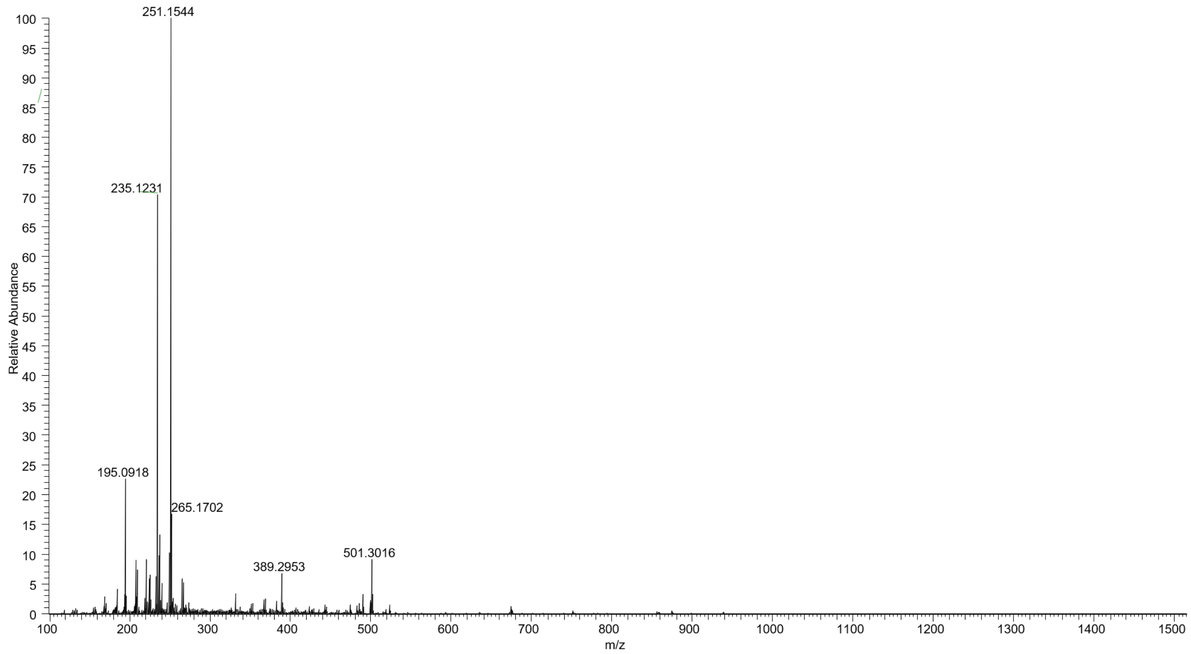

Figure S9 MS spectrum of **1**.

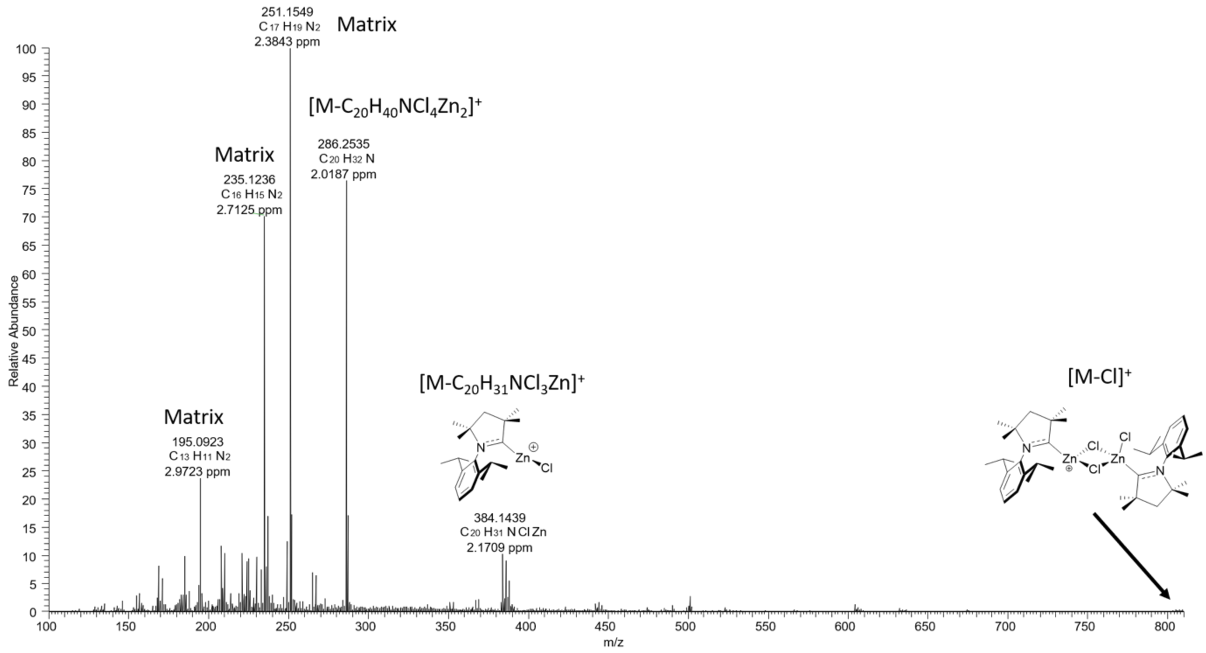

Figure S10 Detail of MS spectrum of **1**.

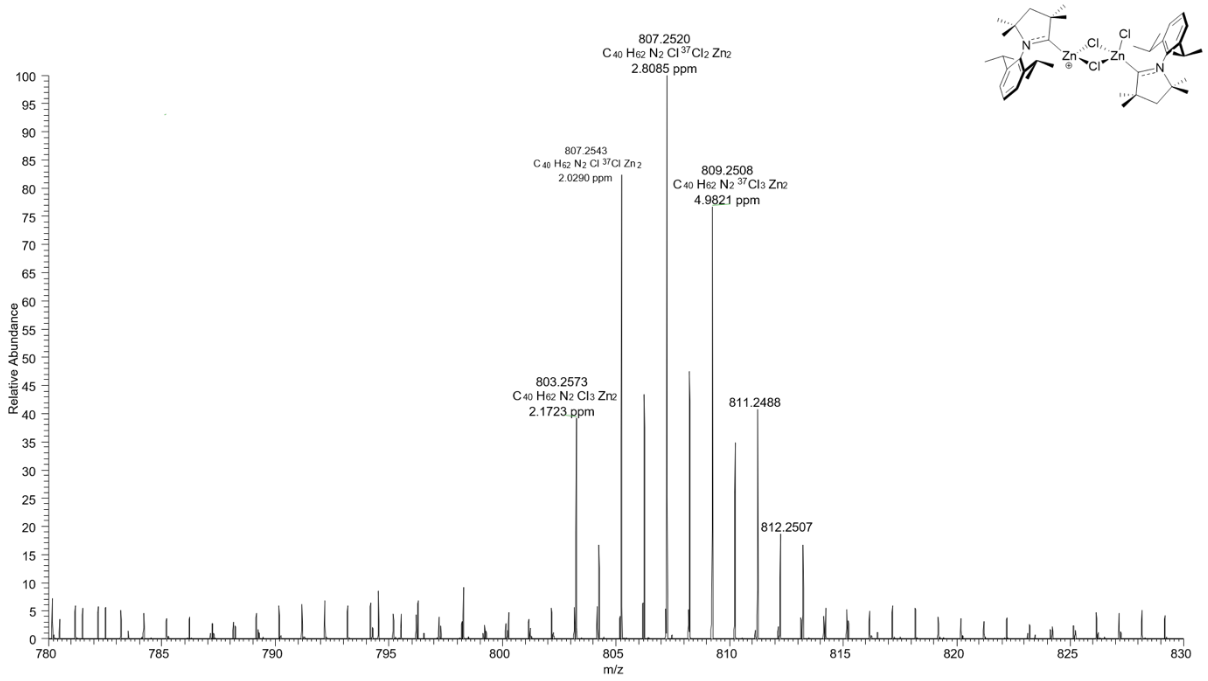

Figure S11 Detail of MS spectrum of **1**.

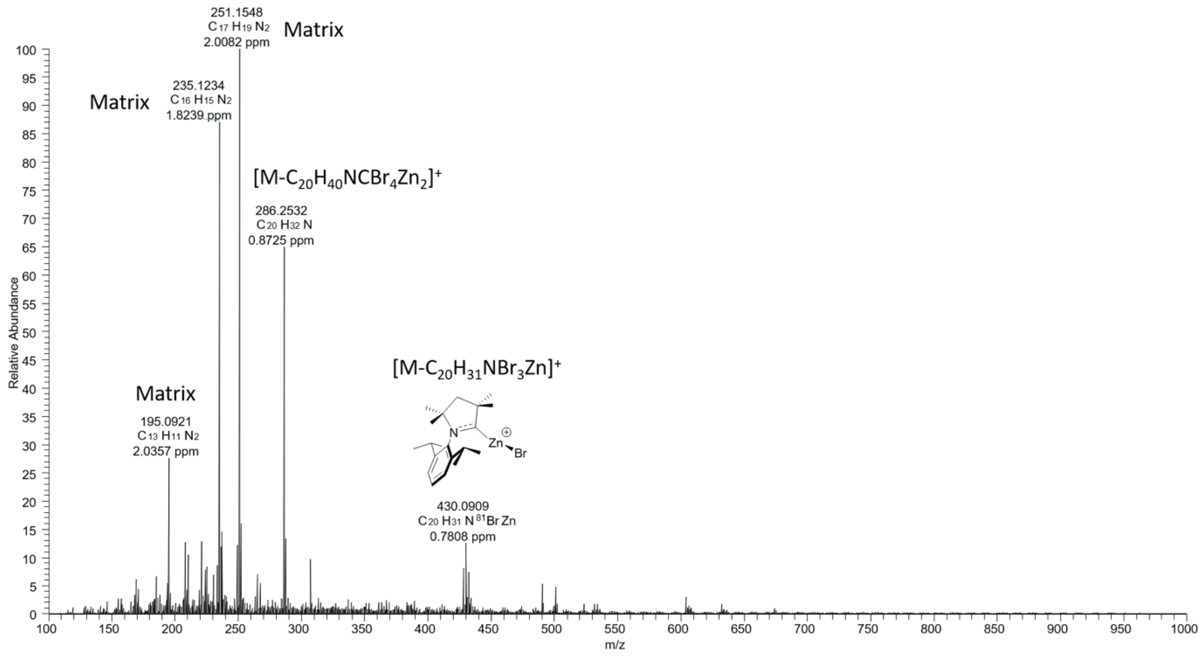

Figure S12 MS spectrum of **2**.

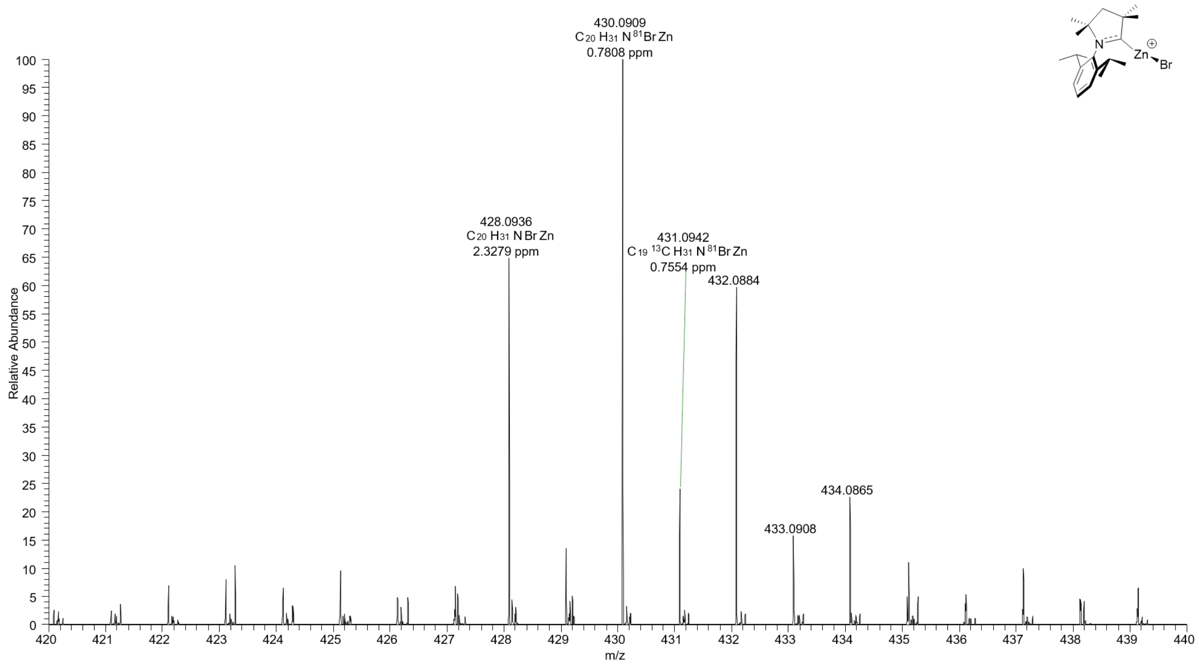

Figure S13 Detail of MS spectrum of **2**.

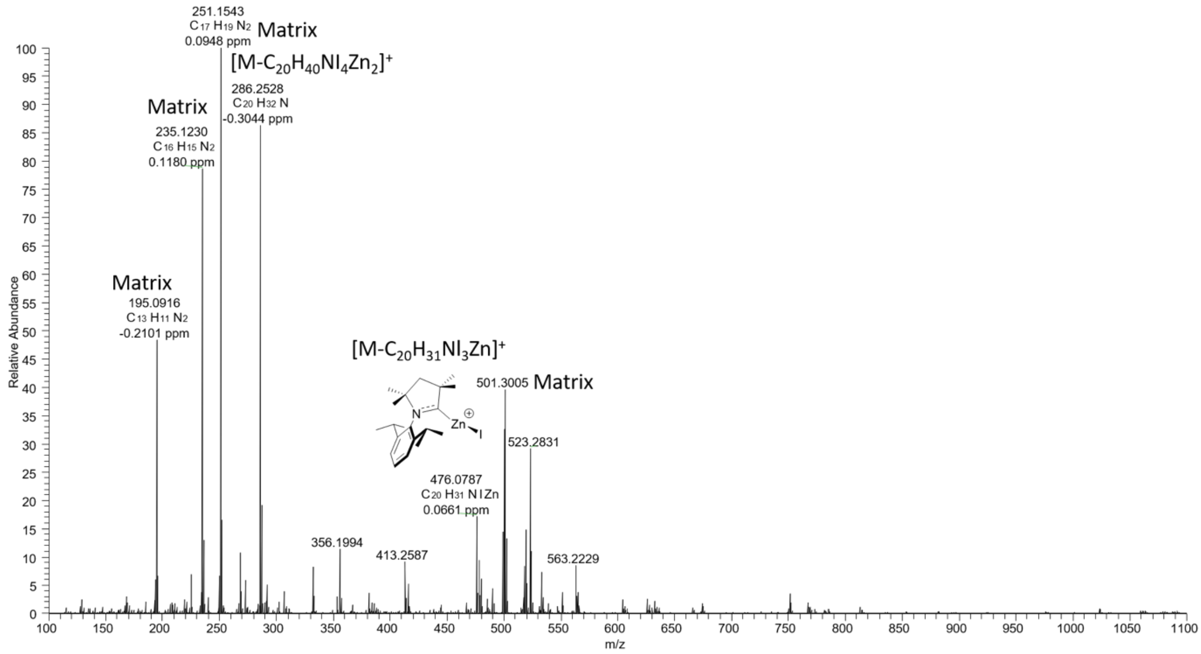

Figure S14 MS spectrum of **3**.

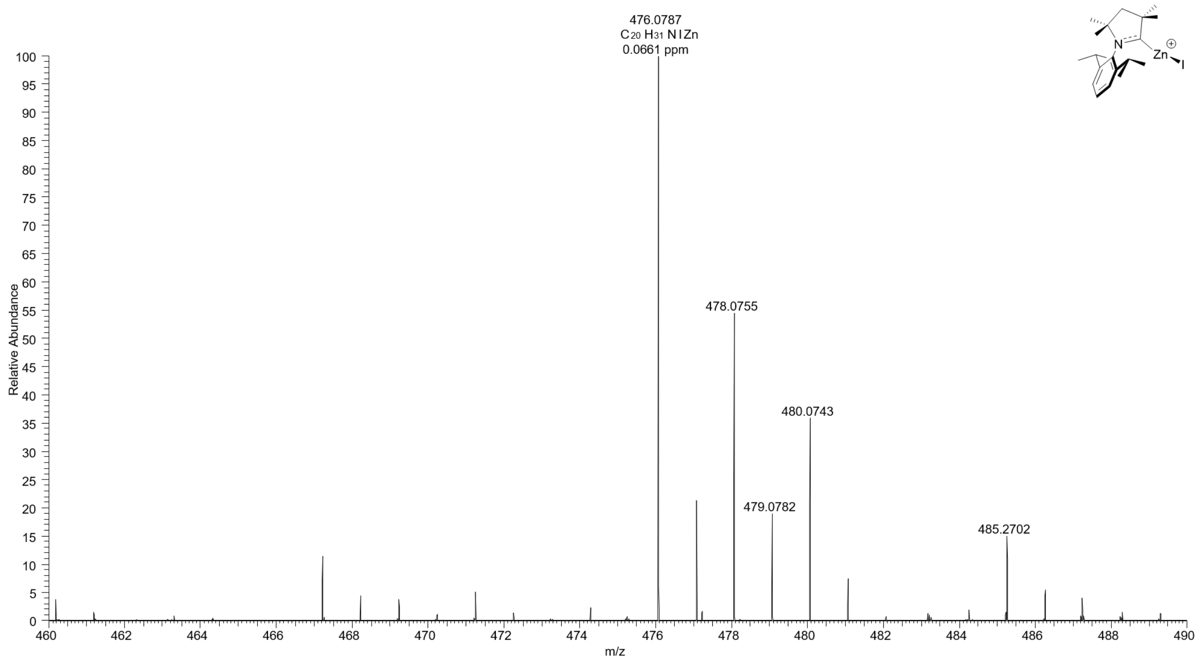

Figure S15 Detail of MS spectrum of **3**.

4. Photophysical measurement

4.1. Absorption spectra

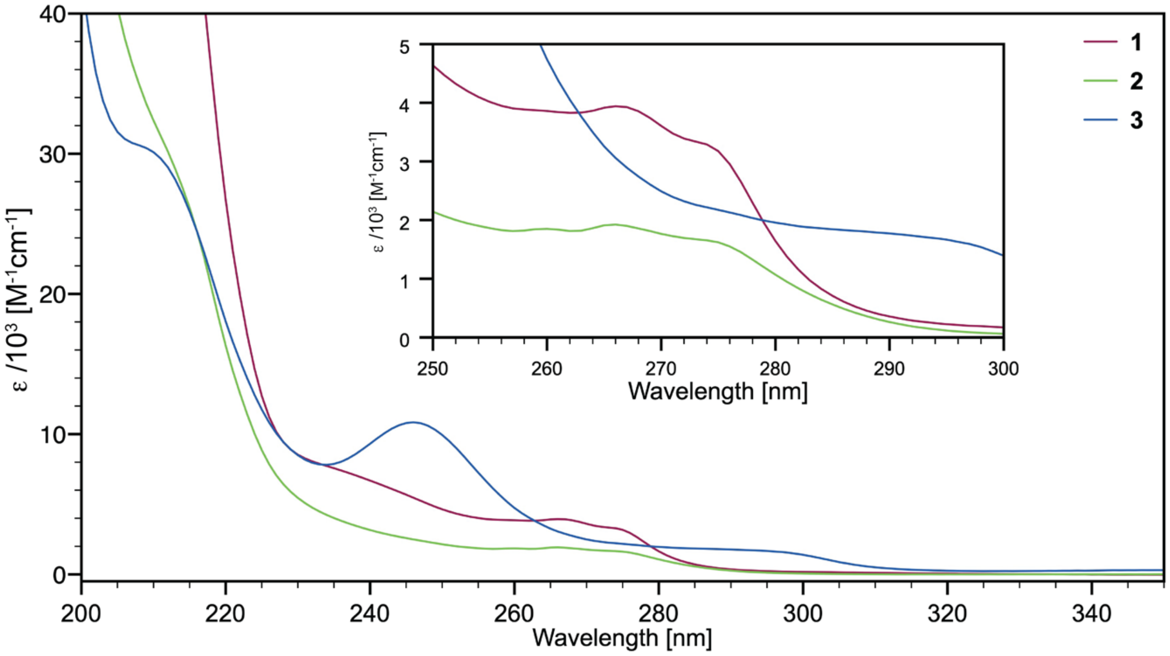

Figure S16 Absorption spectra of **1** - **3** in acetonitrile.

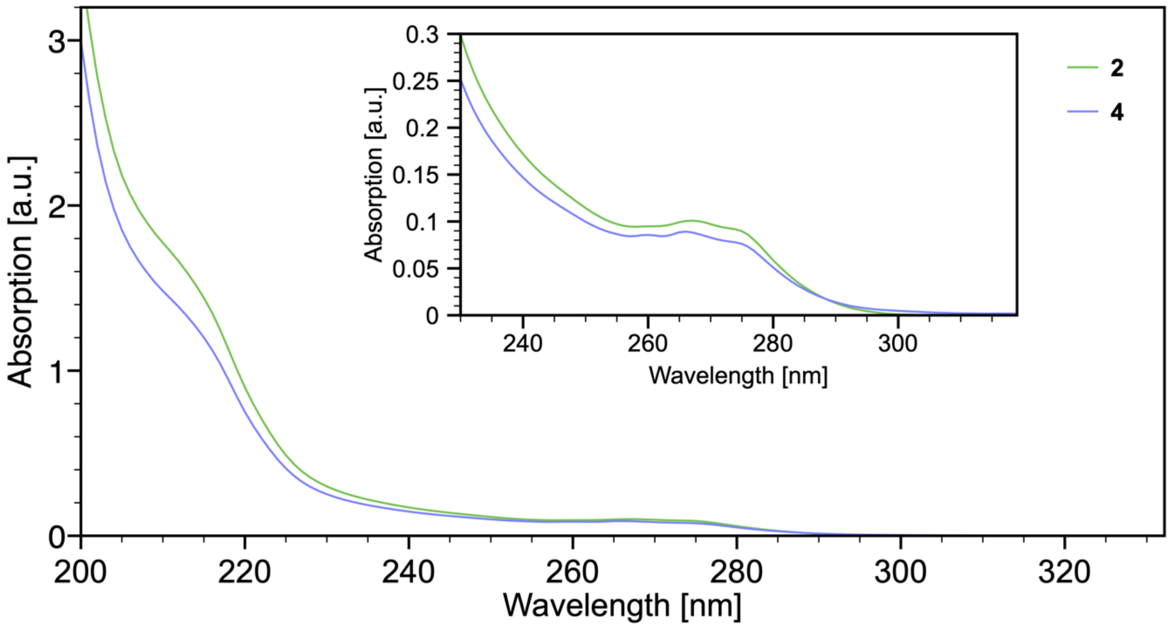

Figure S17 Comparison of absorption spectra of **2** and **4** measured in acetonitrile and using previously isolated dimeric and monomeric species, respectively.

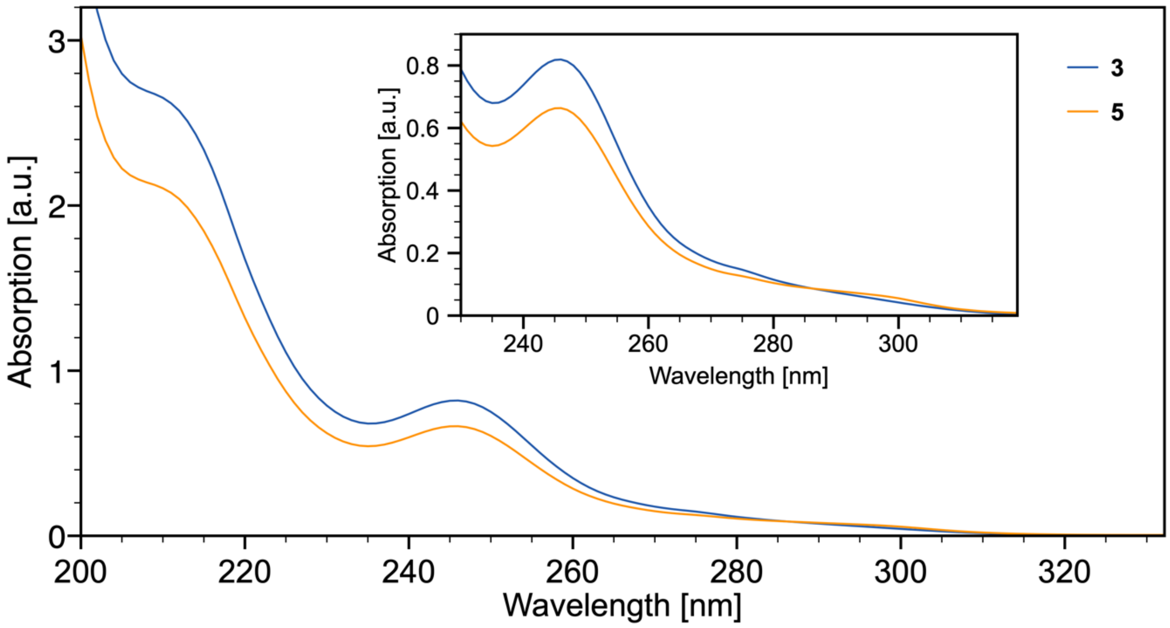

Figure S18 Comparison of absorption spectra of **3** and **5** measured in acetonitrile and using previously isolated dimeric and monomeric species, respectively.

4.2. Measurements of photoinduced transformation in solid state

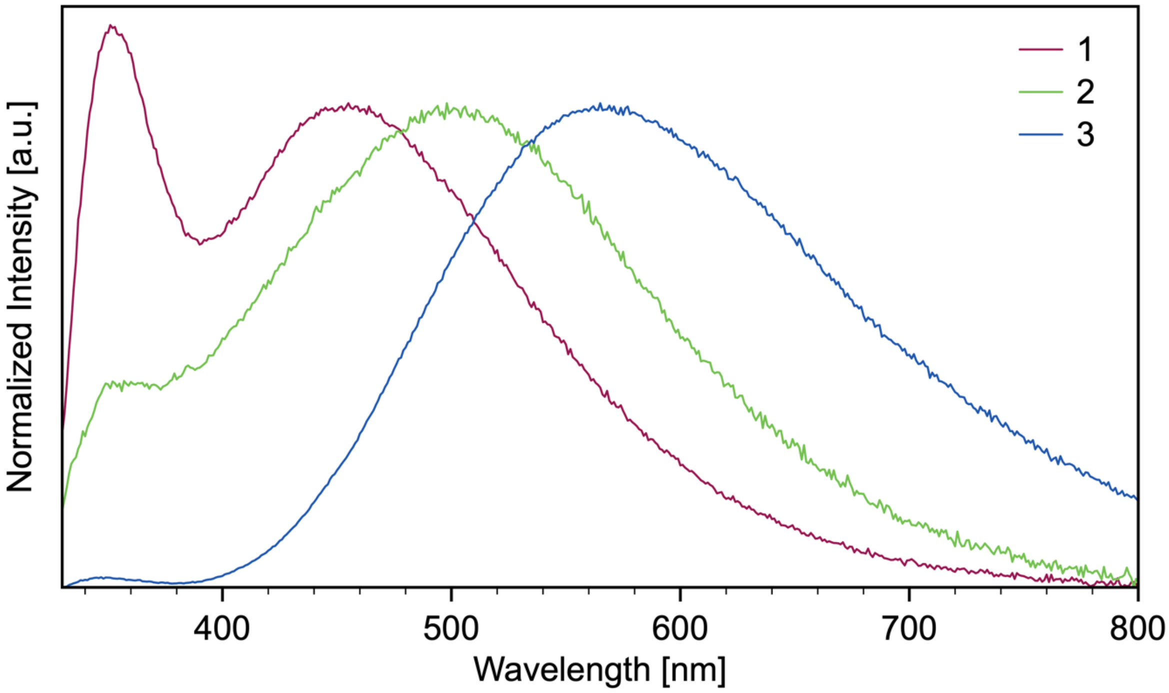

Figure S19 First emission spectra (before pro-longed irradiation of samples) of **1-3**.

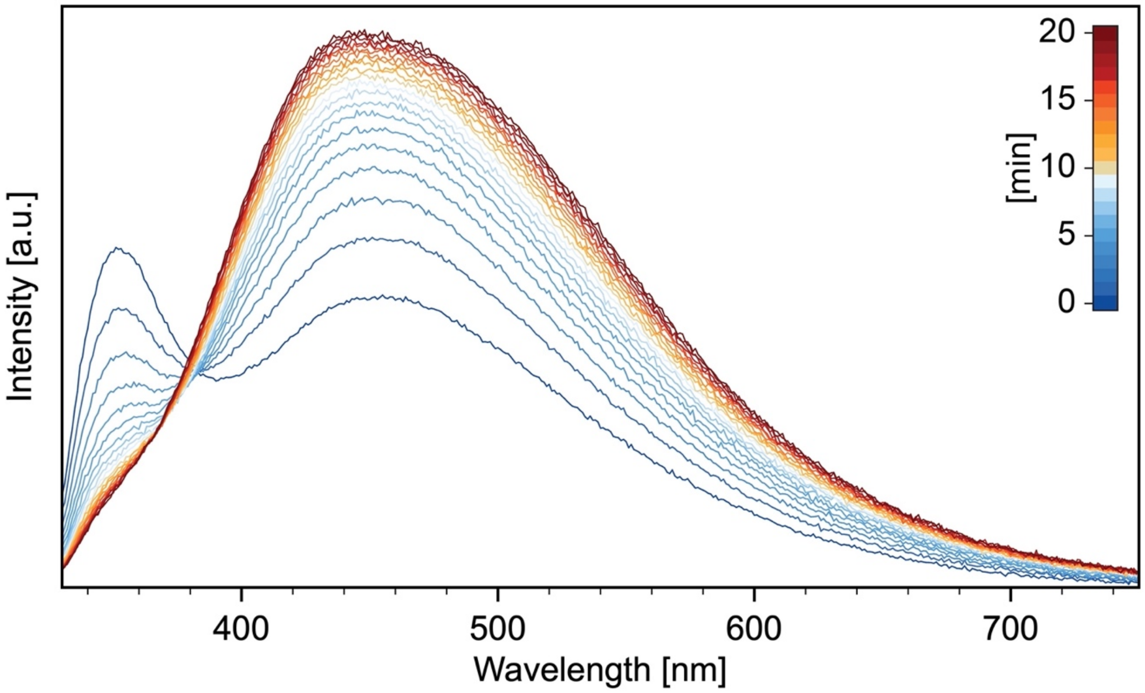

Figure S20 Multiple emission spectra of **1**, recorded with 300 nm excitation, showing progressive evolution of emission profile under UV irradiation.

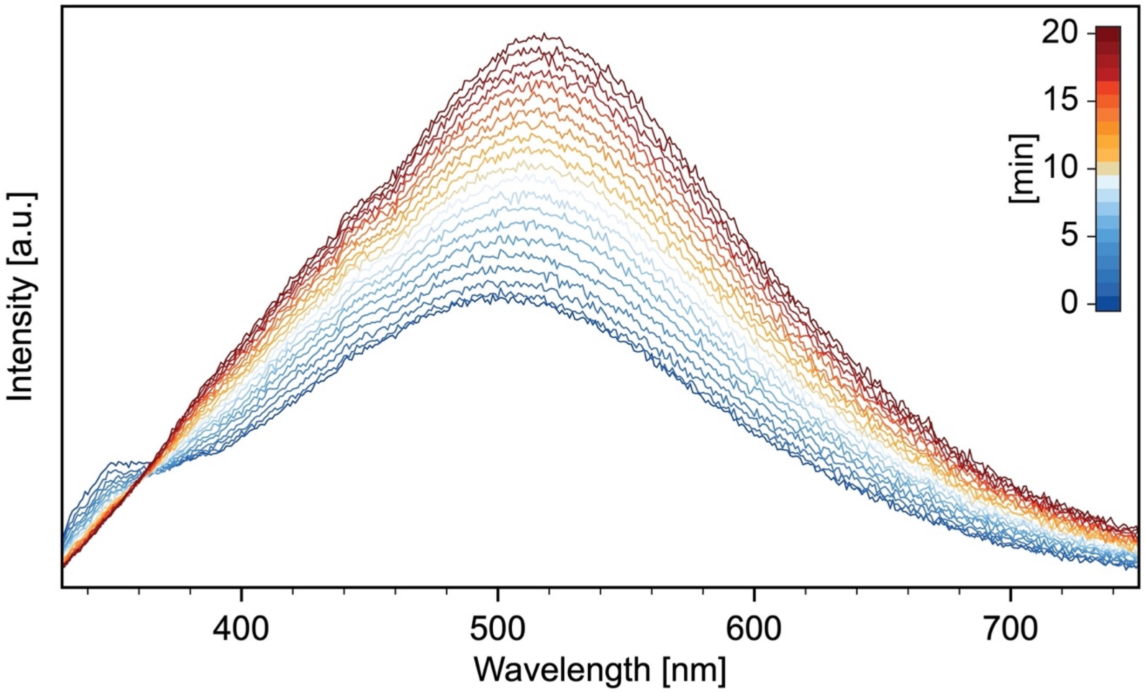

Figure S21 Multiple emission spectra of **2**, recorded with 300 nm excitation, showing progressive evolution of emission profile under UV irradiation.

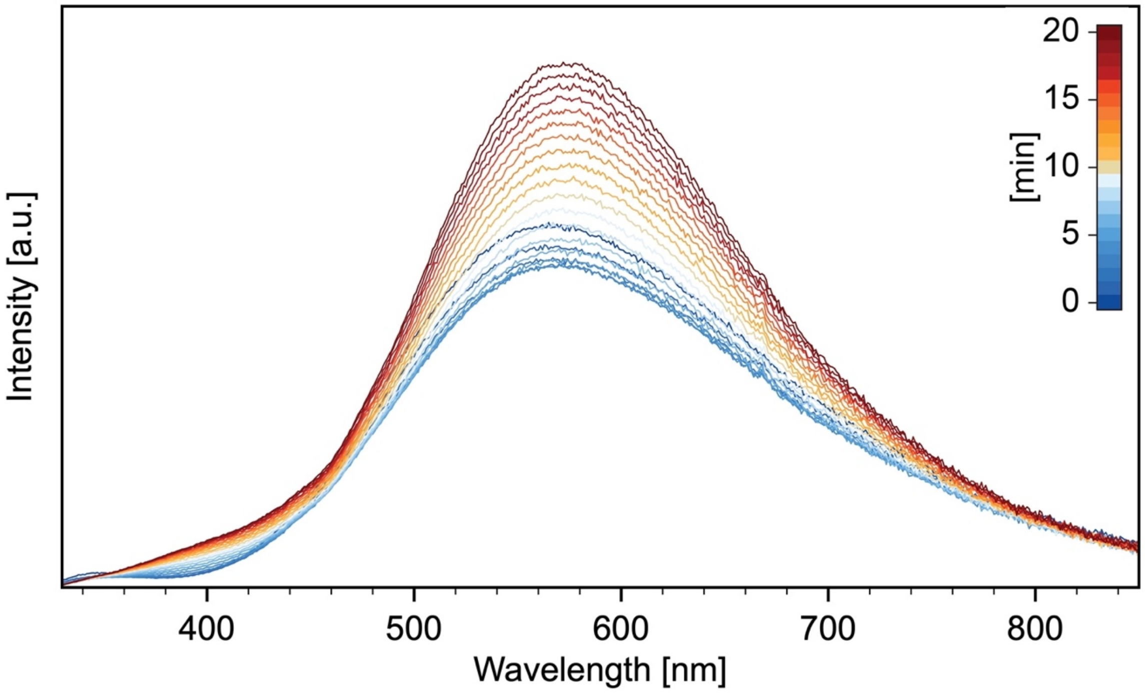

Figure S22 Multiple emission spectra of **3**, recorded with 300 nm excitation, showing progressive evolution of emission profile under UV irradiation.

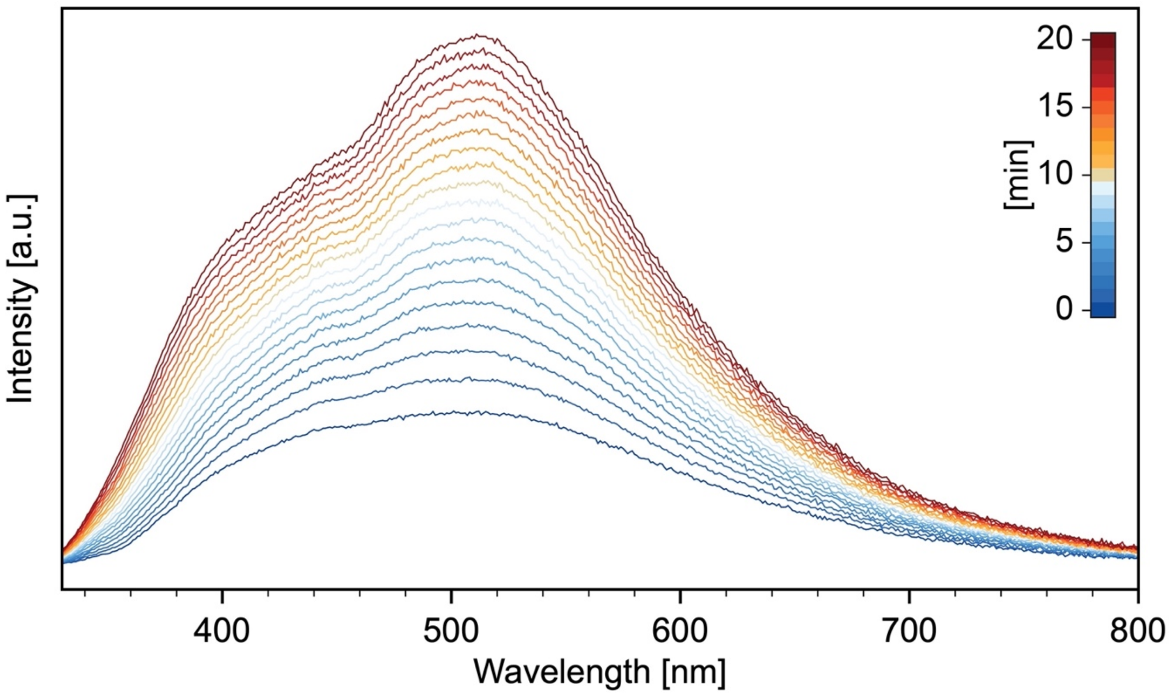

Figure S23 Multiple emission spectra of **4**, recorded with 300 nm excitation, showing progressive evolution of emission profile under UV irradiation.

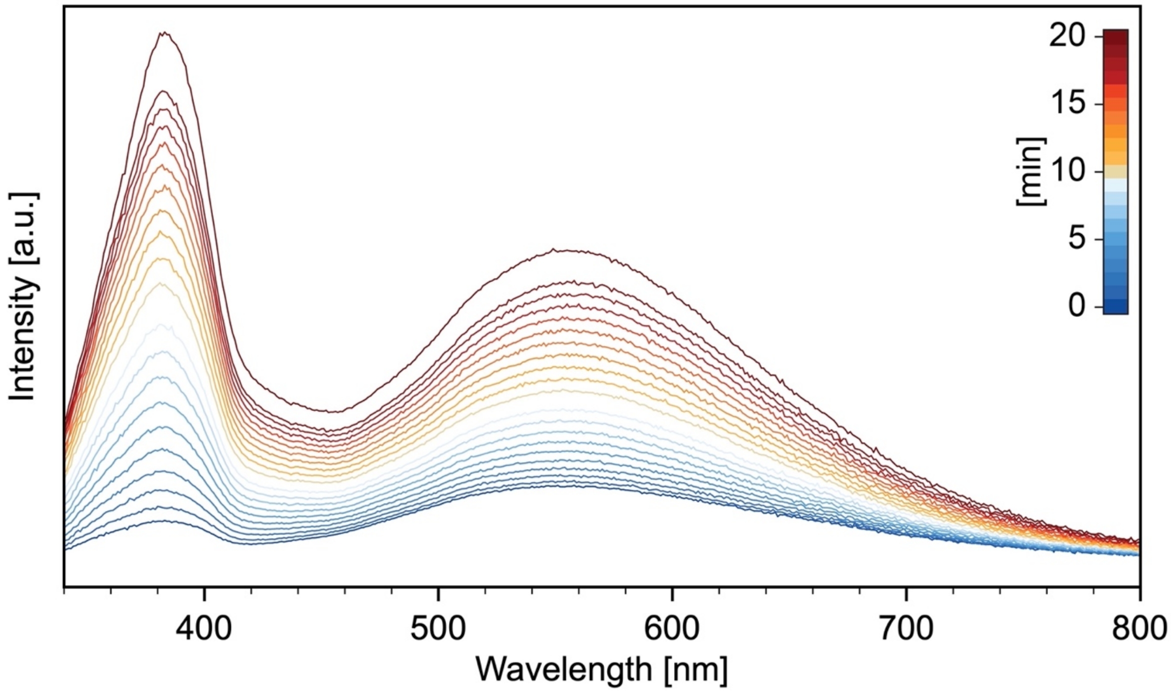

Figure S24 Multiple emission spectra of **5**, recorded with 300 nm excitation, showing progressive evolution of emission profile under UV irradiation.

4.3. Low temperature measurement in solid state

4.3.1. Emission and excitations profiles

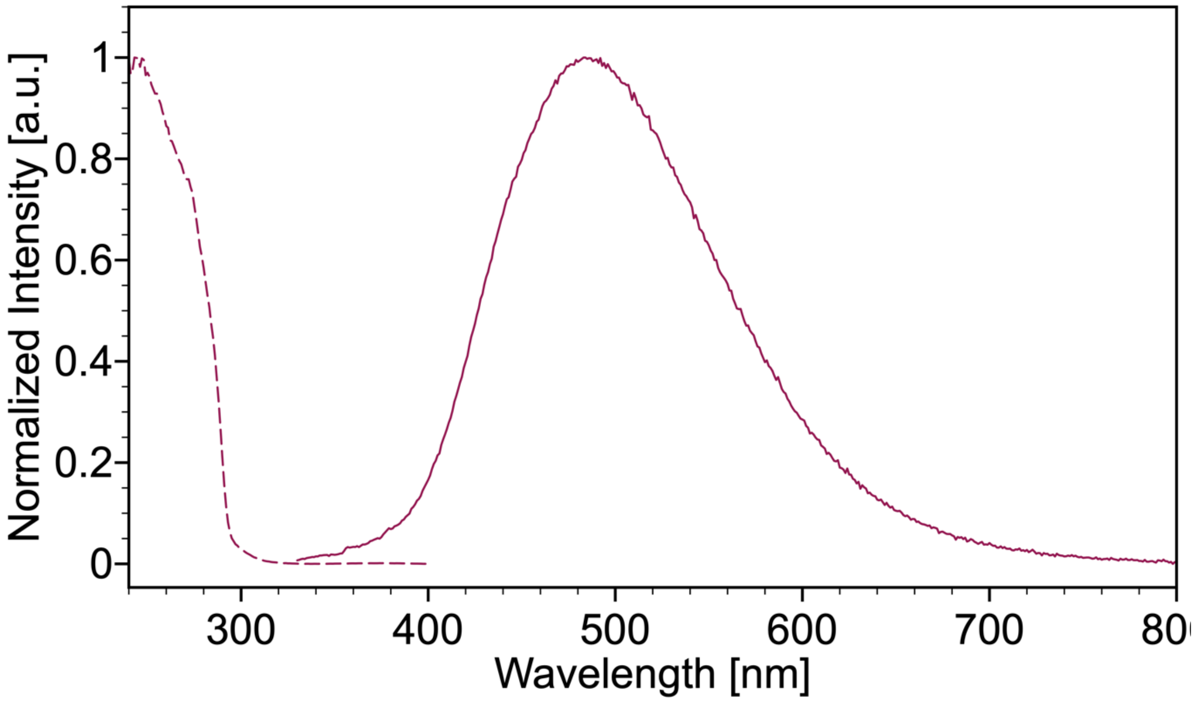

Figure S25 Emission (λ^{Exc} = 300 nm) and excitation (λ^{Em} = 480 nm) spectra of **1**.

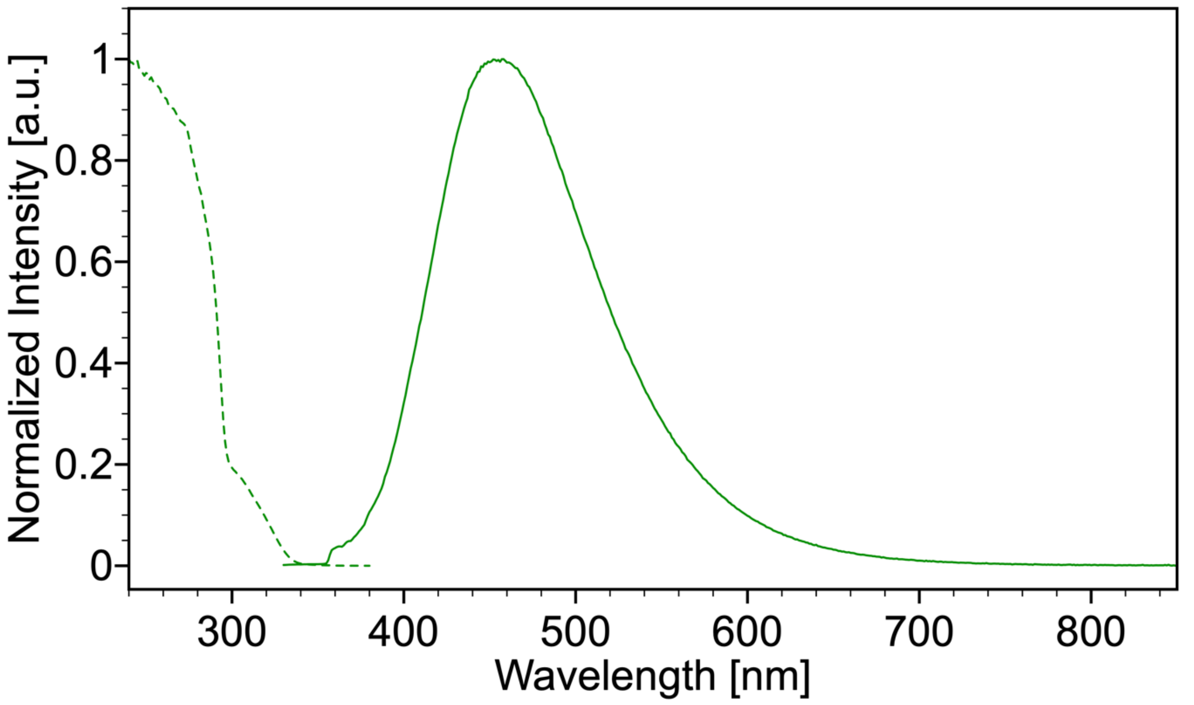

Figure S26 Emission (λ^{Exc} = 300 nm) and excitation (λ^{Em} = 460 nm) spectra of **2**.

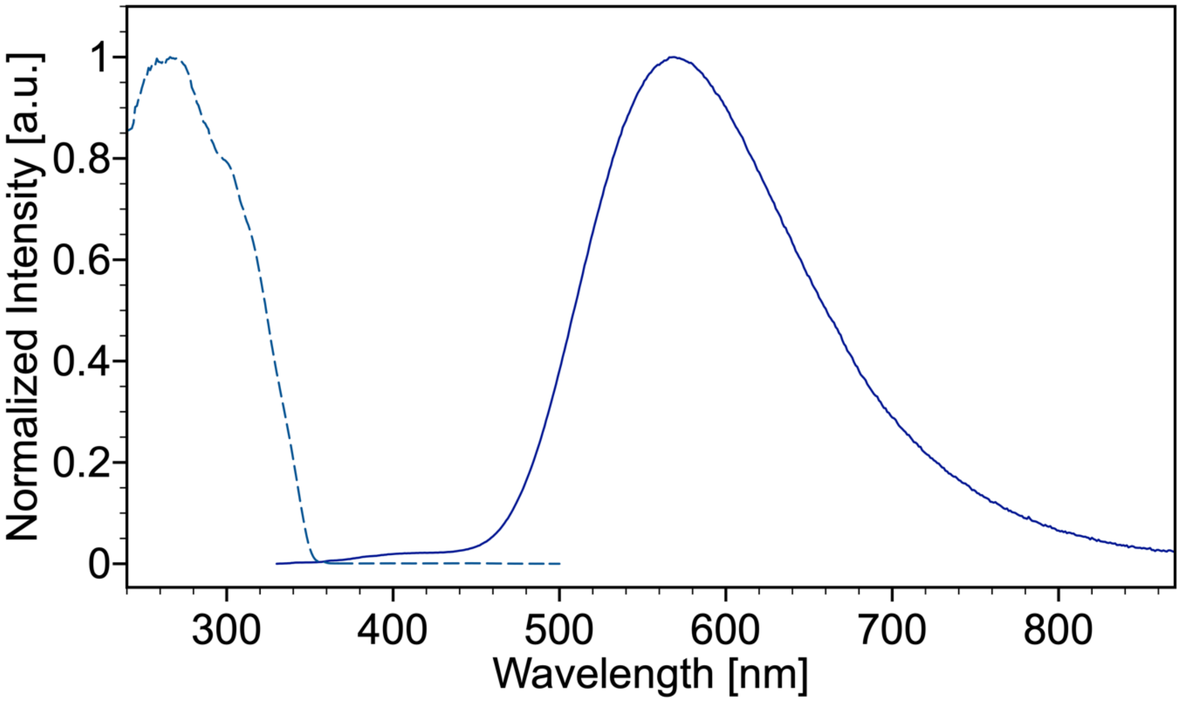

Figure S27 Emission (λ^{Exc} = 310 nm) and excitation (λ^{Em} = 570 nm) spectra of **3**.

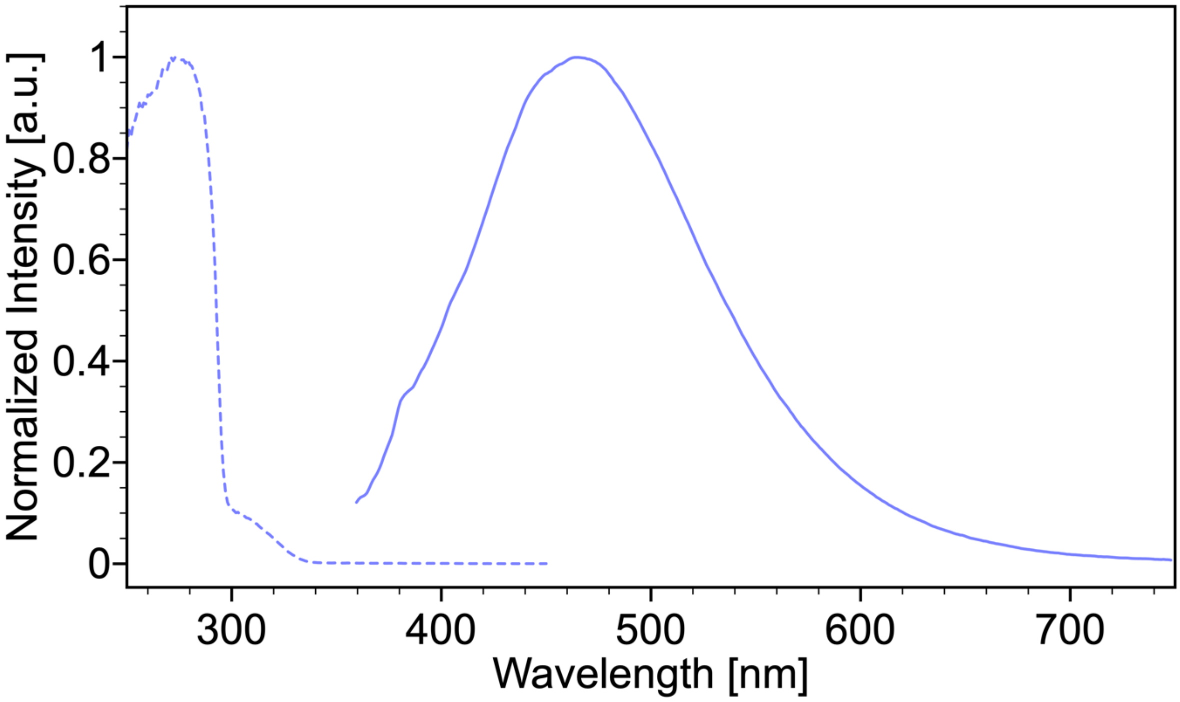

Figure S28 Emission (λ^{Exc} = 290 nm) and excitation (λ^{Em} = 460 nm) spectra of **4**.

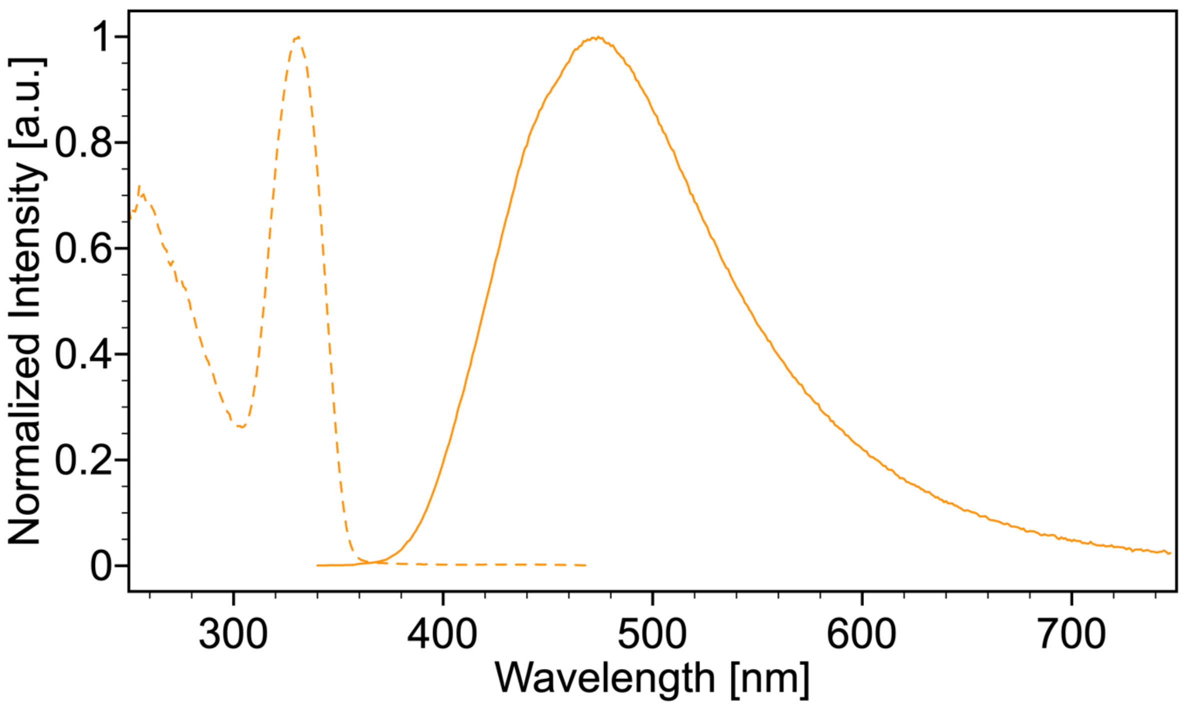

Figure S29 Emission (λ^{Exc} = 330 nm) and excitation (λ^{Em} = 480 nm) spectra of **5**.

4.3.2. Details of lifetime measurements in solid state

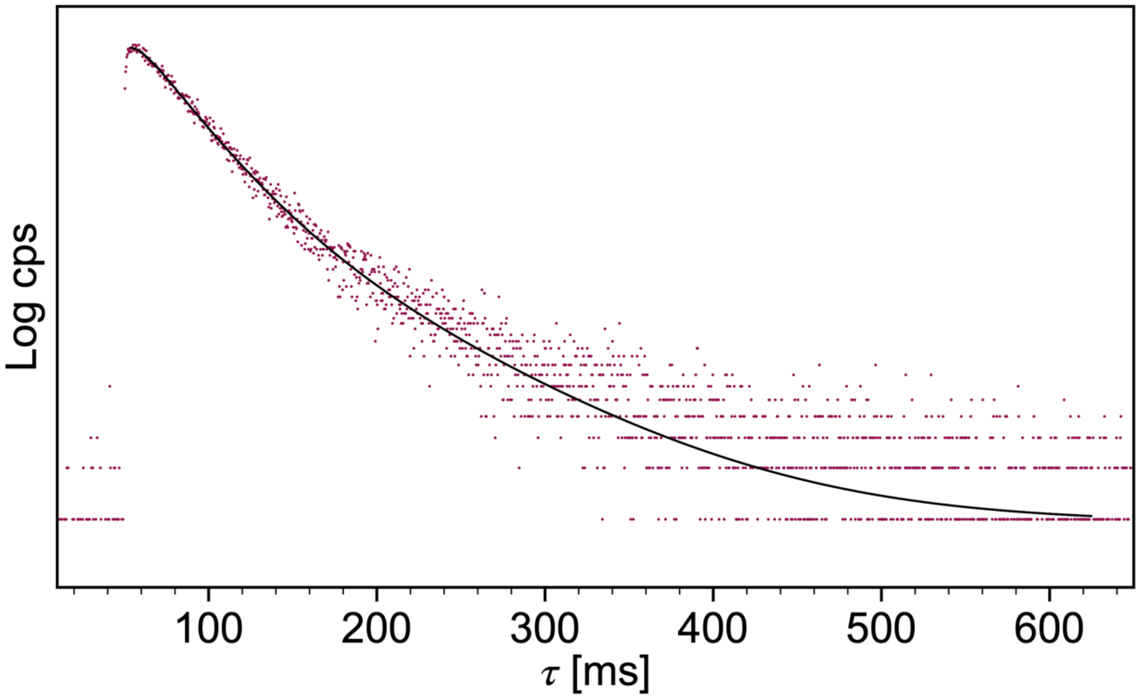

Figure S30 Experimentally obtain (λ^{Exc} = 290 nm, λ^{Em} = 480 nm) luminescence decay (red dots) for **1** and numerical triexponential fit (black).

Table S4 Parameters of triexponential fit of photoluminescence decay of **1** in solid state.

	LT [mS]	pre-exponential factors	χ^2
τ_1	6.98±0.94	-151.2	
τ_2	30.20±0.78	613.1	1.072
τ_2	80.13±2.55	109.3	

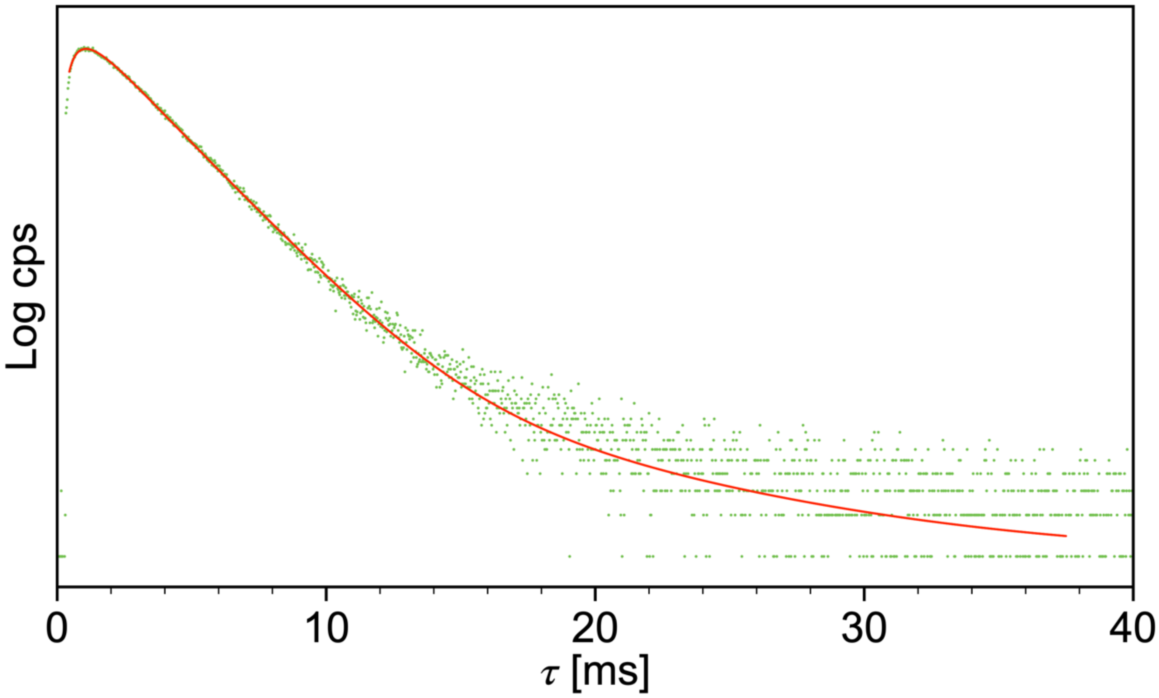

Figure S31 Experimentally obtain (λ^{Exc} = 280 nm, λ^{Em} = 460 nm) luminescence decay (green dots) for **2** and numerical triexponential fit (red).

Table S5 Parameters of triexponential fit of photoluminescence decay of **2** in solid state.

	LT [μS]	pre-exponential factors	χ^2
τ_1	491.12±3.67	-5230.4	
τ_2	2121.60±4.62	8490.5	1.168
τ_2	7597.20±349.23	54.43	

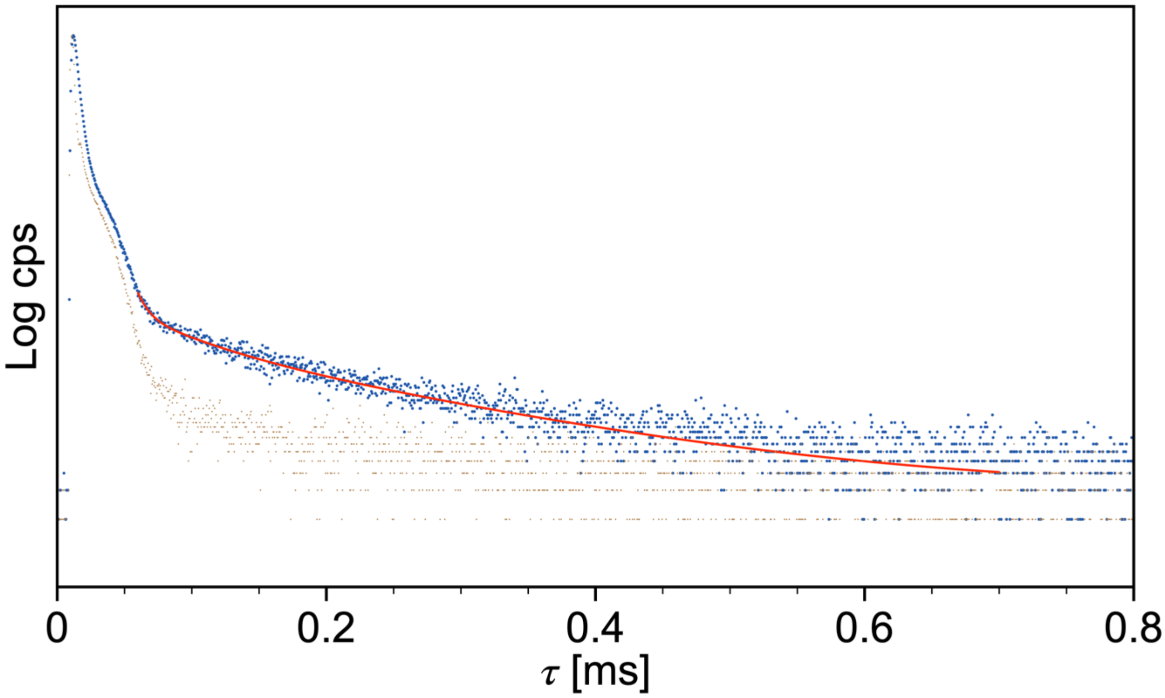

Figure S32 Experimentally obtain (λ^{Exc} = 310 nm, λ^{Em} = 570 nm) luminescence decay (blue dots) for **3**, numerical triexponential fit (red) and IRF measurement (brown dots).

Table S6 Parameters of triexponential fit of photoluminescence decay of **3** in solid state.

	**LT [μS]**	**pre-exponential factors**	χ^2
τ_1	6.96±0.90	96.1	
τ_2	44.79±7.04	62.1	1.158
τ_2	155.05±9.89	62.5	

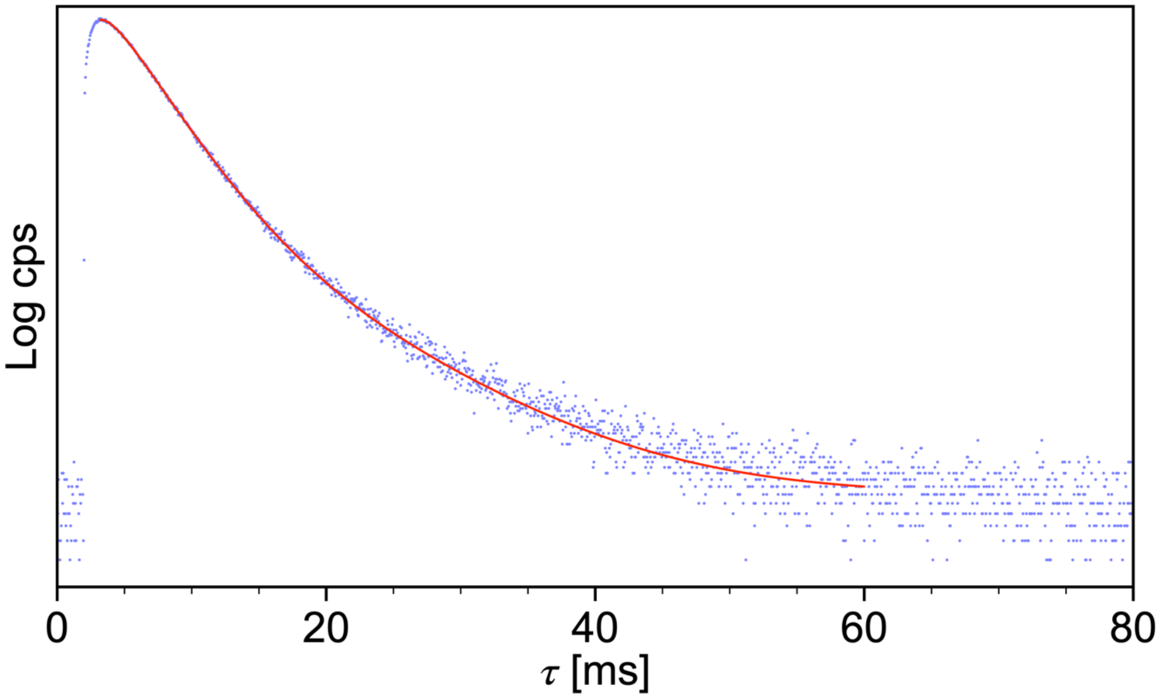

Figure S33 Experimentally obtain (λ^{Exc} = 280 nm, λ^{Em} = 460 nm) luminescence decay (blue dots) for **4** and numerical triexponential fit (red).

Table S7 Parameters of triexponential fit of photoluminescence decay of **4** in solid state.

	**LT [μS]**	**pre-exponential factors**	χ^2
τ_1	687.78±18.25	-4803.8	
τ_2	3014.88±15.69	12597.0	1.116
τ_2	8314.44±86.59	993.2	

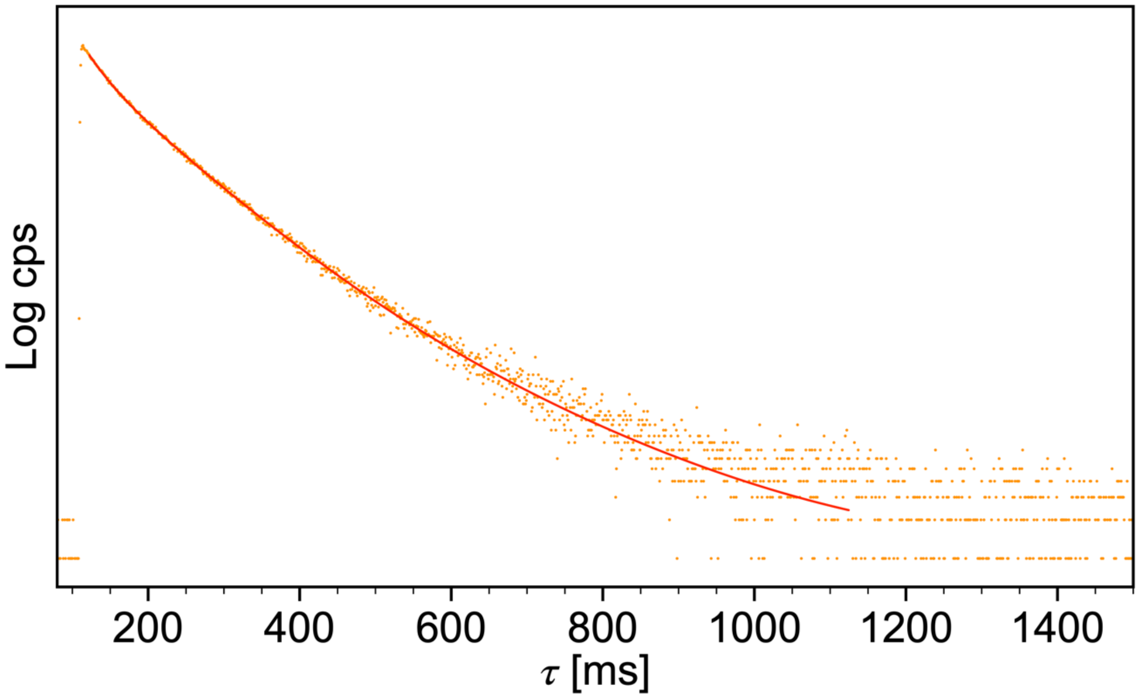

Figure S34 Experimentally obtain (λ^{Exc} = 330 nm, λ^{Em} = 475 nm) luminescence decay (orange dots) for **5** and numerical triexponential fit (red).

Table S7 Parameters of triexponential fit of photoluminescence decay of **5** in solid state.

	LT [μS]	pre-exponential factors	χ^2
τ_1	25.22±0.71	2457.0	
τ_2	80.83±1.22	5478.9	1.129
τ_2	169.51±8.25	449.2	

5. Computational details

Table S8. Adiabatic DFT/MRCI energies (values in parentheses include SOC at the QDPT level) of the lowest ungerade singlet and triplet states of the dimers in a mildly polar environment (ε = 4.711), vertical emission wavelengths and averaged phosphorescence lifetimes of the lowest triplet state at the respective molecular geometry. * denotes symmetry-broken localized solutions.

		Geometry				
	State	S_0<br>(^1A_g)	^{1}XCT/LE$_{caac}$<br>(^1A_u)	^{3}XCT/LE$_{caac}$<br>(^3A_u)	^{3}LE$_{dipp}$<br>(^3A_u)	^{3}LE$_{dipp}$<br>(^3A)*
**1**	S_0	0.00 eV	0.27 eV	0.30 eV	0.13 eV	0.93 eV
	^{1}XCT/LE$_{caac}$	4.53 eV	4.30 eV	4.32 eV	4.61 eV	5.24 eV
	^{3}XCT/LE$_{caac}$	3.90 eV	3.67 eV	3.66 eV<br>369 nm<br>10 ms	3.99 eV	4.76 eV
	^{3}LE$_{dipp}$	3.75 eV	4.00 eV	4.03 eV	3.54 eV<br>364 nm<br>1 s	3.50 eV<br>482 nm<br>3 s
**2**	S_0	0.00 eV	0.25 eV	0.28 eV	0.15 eV	0.83 eV
	^{1}XCT/LE$_{caac}$	4.48 eV<br>(4.47 eV)	4.25 eV<br>(4.25 eV)	4.26 eV<br>(4.26 eV)	4.58 eV<br>(4.57 eV)	5.13 eV<br>(5.13 eV)
	^{3}XCT/LE$_{caac}$	3.89 eV<br>(3.89 eV)	3.65 eV<br>(3.65 eV)	3.63 eV<br>(3.63 eV)<br>372 nm<br>640 μs	3.97 eV<br>(3.97 eV)	4.63 eV<br>(4.63 eV)
	^{3}LE$_{dipp}$	3.80 eV<br>(3.80 eV)	4.05 eV<br>(4.05 eV)	4.06 eV<br>(4.06 eV)	3.59 eV<br>(3.59 eV)<br>360 nm<br>47 ms	3.47 eV<br>(3.47 eV)<br>469 nm<br>79 ms
**3**	S_0	0.00 eV	0.40 eV	0.29 eV		0.79 eV
	^{1}XCT/LE$_{caac}$	4.19 eV<br>(4.08 eV)	3.87 eV<br>(3.86 eV)	4.06 eV<br>(4.00 eV)		4.71 eV<br>(4.63 eV)
	^{3}XCT/LE$_{caac}$	3.75 eV<br>(3.71 eV)	3.83 eV<br>(3.68 eV)	3.48 eV<br>(3.47 eV)<br>393 nm<br>27 μs		4.41 eV<br>(4.39 eV)
	^{3}LE$_{dipp}$	3.82 eV<br>(3.83 eV)	4.18 eV<br>(4.18 eV)	4.07 eV<br>(4.09 eV)		3.43 eV<br>(3.44 eV)<br>470 nm<br>2 ms

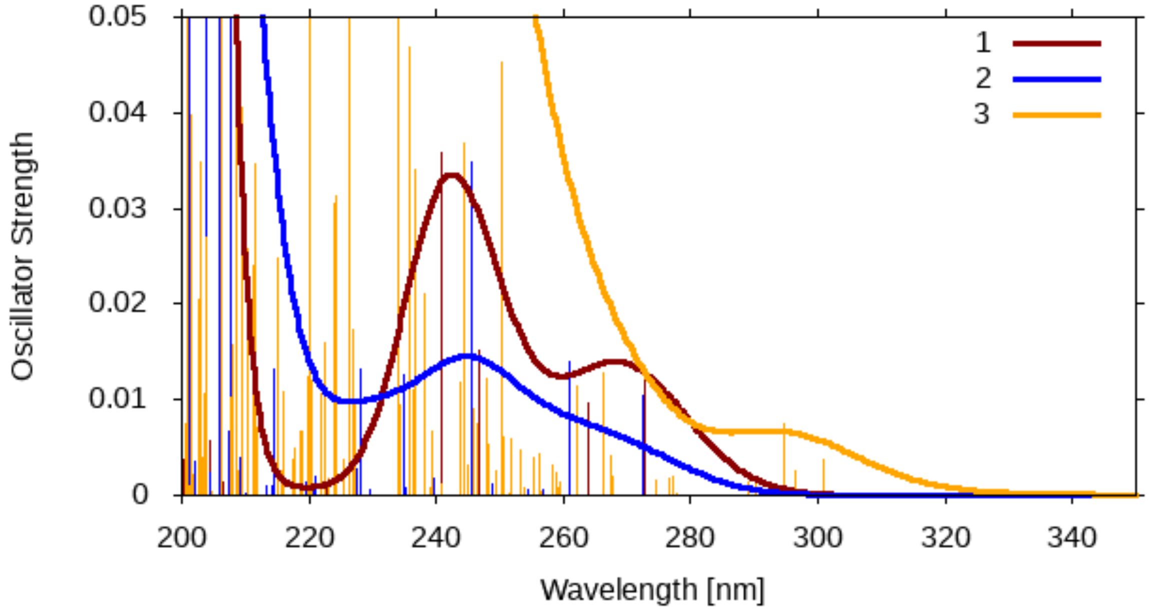

Figure S35 Calculated absorption spectra of **1** - **3**.

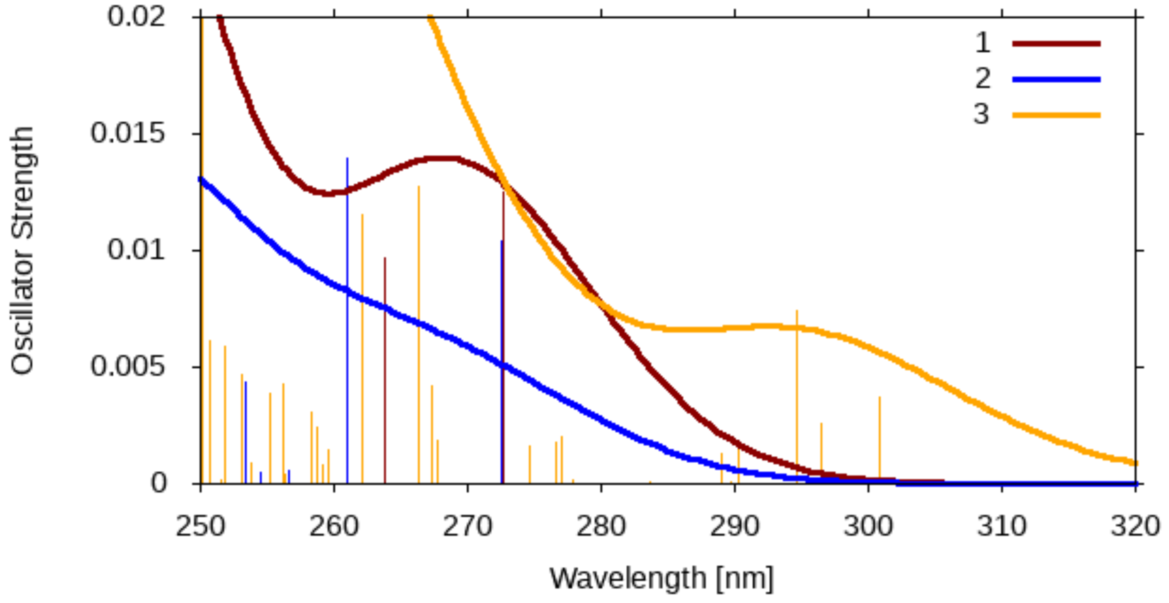

Figure S36 Calculated absorption spectra of **1** - **3** (detail).

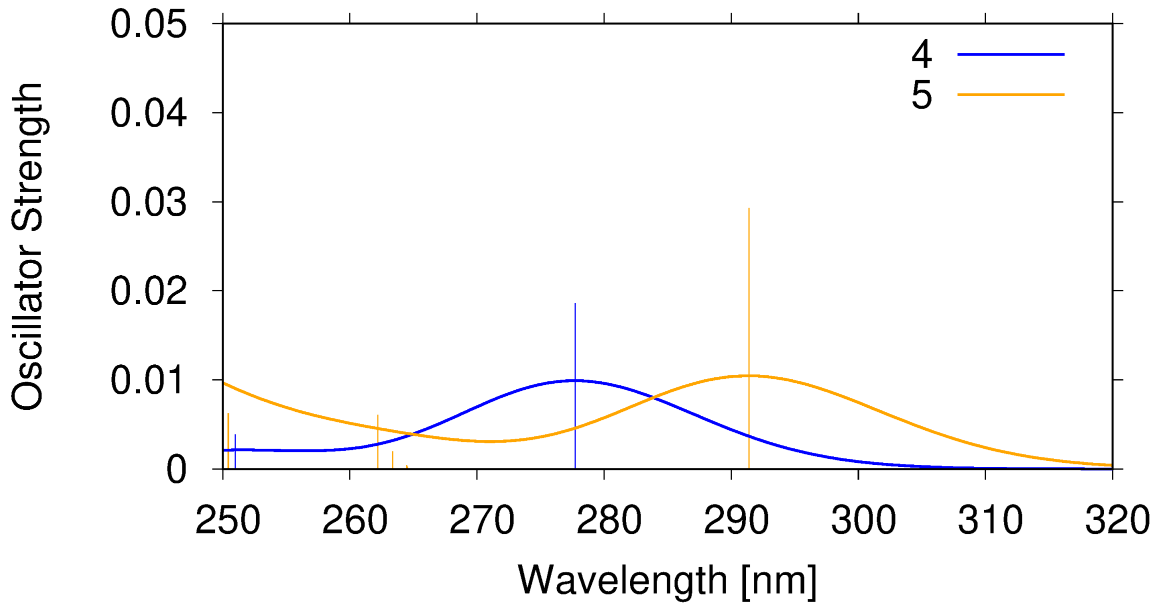

Figure S37 Calculated absorption spectra of **4** and **5**.

6. References

[1] G. M. Sheldrick, *Acta Crystallographica Section A* **2015**, *71*, 3–8.
[2] G. M. Sheldrick, *Acta Crystallographica Section C* **2015**, *71*, 3–8.
[3] O. v Dolomanov, L. J. Bourhis, R. J. Gildea, J. A. K. Howard, H. Puschmann, *Journal of Applied Crystallography* **2009**, *42*, 339–341.
[4] A. L. Spek, *Acta Crystallographica Section C* **2015**, *71*, 9–18.
